# The role of strigolactones in P deficiency induced transcriptional changes in tomato roots

**DOI:** 10.1186/s12870-021-03124-0

**Published:** 2021-07-23

**Authors:** Yanting Wang, Hernando G. Suárez Duran, Jan C. van Haarst, Elio G.W.M Schijlen, Carolien Ruyter-Spira, Marnix H. Medema, Lemeng Dong, Harro J. Bouwmeester

**Affiliations:** 1 Plant Hormone Biology Group, Swammerdam Institute for Life Sciences, University of Amsterdam, Amsterdam, The Netherlands; 2 Bioinformatics Group, Wageningen University and Research, Wageningen, The Netherlands; 3 Business Unit Bioscience, Plant Research International, Wageningen, The Netherlands; 4 Laboratory of Plant Physiology, Wageningen University and Research, Wageningen, The Netherlands

**Keywords:** P starvation, Tomato, Root, Strigolactone, RNAseq, Transcriptional changes

## Abstract

**Background:**

Phosphorus (P) is an essential macronutrient for plant growth and development. Upon P shortage, plant responds with massive reprogramming of transcription, the Phosphate Starvation Response (PSR). In parallel, the production of strigolactones (SLs)—a class of plant hormones that regulates plant development and rhizosphere signaling molecules—increases. It is unclear, however, what the functional link is between these two processes. In this study, using tomato as a model, RNAseq was used to evaluate the time-resolved changes in gene expression in the roots upon P starvation and, using a tomato *CAROTENOID CLEAVAGE DIOXYGENASES 8* (*CCD8*) RNAi line, what the role of SLs is in this.

**Results:**

Gene ontology (GO)-term enrichment and KEGG analysis of the genes regulated by P starvation and P replenishment revealed that metabolism is an important component of the P starvation response that is aimed at P homeostasis, with large changes occurring in glyco-and galactolipid and carbohydrate metabolism, biosynthesis of secondary metabolites, including terpenoids and polyketides, glycan biosynthesis and metabolism, and amino acid metabolism. In the *CCD8* RNAi line about 96% of the PSR genes was less affected than in wild-type (WT) tomato. For example, phospholipid biosynthesis was suppressed by P starvation, while the degradation of phospholipids and biosynthesis of substitute lipids such as sulfolipids and galactolipids were induced by P starvation. Around two thirds of the corresponding transcriptional changes depend on the presence of SLs. Other biosynthesis pathways are also reprogrammed under P starvation, such as phenylpropanoid and carotenoid biosynthesis, pantothenate and CoA, lysine and alkaloids, and this also partially depends on SLs. Additionally, some plant hormone biosynthetic pathways were affected by P starvation and also here, SLs are required for many of the changes (more than two thirds for Gibberellins and around one third for Abscisic acid) in the gene expression.

**Conclusions:**

Our analysis shows that SLs are not just the end product of the PSR in plants (the signals secreted by plants into the rhizosphere), but also play a major role in the regulation of the PSR (as plant hormone).

**Supplementary Information:**

The online version contains supplementary material available at 10.1186/s12870-021-03124-0.

## Background

Phosphorus (P) plays an important role in various processes of plant growth and development [1]. However, P is usually the least available of all essential nutrients in the soil due to complexation and slow diffusion [2], and the continued high application of P fertilizer is not sustainable [3]. Therefore, improving P use efficiency of plants is of vital importance. To achieve this, a better understanding of the mechanisms by which plants deal with low P availability is essential.

To adapt to low P availability, plants have evolved physiological, biochemical and spatio-temporal molecular responses aimed at acquiring more P from the environment and remobilizing P from structures and processes where they are least required [1, 4, 5].

- (1) Inhibition of primary root growth and enhanced lateral root formation are characteristic for the changes in root architecture in response to P starvation [6]. This acclimation enlarges the root surface area and hence enhances the possibility of roots acquiring more P from the soil [6].
- (2) A biochemical strategy for increasing P acquisition from the soil is the secretion of organic acids and extracellular acid phosphatases (APs) by the roots into the soil [5]. Inorganic P generally is complexed by metal ions but becomes available to the plant when it is solubilized by organic anions or H^+^ [5]. Similarly, organic P only becomes available to plants when it is hydrolyzed into free P by phosphatases/phytases, such as PURPLE ACID PHOSPHATASE (PAP) [5]. In many species, genes like *ACID PHOSPHATASE* (*AP*) and *PAP* are induced by P starvation, such as *Arabidopsis APs* (*AtAPs*) and white lupin (*Lupinus albus*) *SECRETORY AP 2* (*LaSAP2*).
- (3) P transporters play an important role in P acquisition and reallocation [5]. When the concentration of P in the growth medium of *Arabidopsis* is low, the expression of the high-affinity P transporter, *PHOSPHATE TRANSPORTER 1* (*PHT1*), is induced, and the corresponding protein accumulates [7]. The importance of the induction of *PHTs* for P uptake has been demonstrated in several species like rice, wheat and soybean [8–11].
- (4) Symbiosis with arbuscular mycorrhizal fungi (AMF) is a strategy used by around 70–80% of land plants to cope with P deficiency [12]. The advantage of this P uptake strategy is that AMF scavenge large volumes of soil for P which they transfer directly to the root cortical cells [12].
- (5) Under P starvation, intracellular APs are induced, which primarily play a role in internal P remobilization by releasing P from senescing tissues and redirecting carbon metabolism to avoid P requiring carbon metabolism [1]. The expression of *AtAP5* in senescent tissues is strongly induced by P starvation but there is no evidence for secretion of *AtAP5*, suggesting that AP5 is more involved in P remobilization within the plants than P acquisition from the soil [1]. AP from Tomato, P starvation-induced gene, (*LePS2)* has also been characterized to be involved in internal P remobilization [13].

It has been shown that hormones play an important role in the acclimation of plants to nutrient deficiencies [14]. Under P deficiency, the production of ethylene increases [15]. P starvation represses both the level of cytokinins (CK) and CK signaling [16]. P deficiency has been shown to repress the level of bioactive gibberellins, which results in the accumulation of DELLA, a repressor of the GA signaling pathway [15, 16]. Moreover, in *Arabidopsis* and barley roots, the level of ABA is elevated under P deficiency [16, 17].

The upregulation of strigolactones (SLs) under P starvation plays a role in the acclimation of shoot and root architecture to low P [18–20]. The increasing number of hormonal signaling roles that are reported for the SLs prompted us to investigate whether SLs also play a role in the many other P-starvation-induced responses in plants, including—and/or partially through—the remodeling of the metabolism of other plant hormones. To investigate this, we followed an untargeted approach and studied the dynamics of the transcriptional changes under P starvation, and the effect of SLs on this process as possible mediators of the Phosphate Starvation Response (PSR). We did this study using tomato, in which the production of SLs is strongly upregulated under P deficiency [21] and which displays a strong PSR. The latter includes induction of the expression of PSR genes such as tomato *AP*, *PHT* and *SPX DOMAIN CONTAINING PROTEINS (SPX)*, the secretion of AP, and an increase in the root: shoot ratio [7, 13, 22–26]. To investigate the role of SLs in this PSR, we analyzed the transcriptional changes in tomato roots in a comprehensive P starvation and P resupply time-course in wild-type (WT) and a transgenic *CAROTENOID CLEAVAGE DIOXYGENASES 8* (*CCD8*) RNAi knockdown line with strongly reduced SL production. We show that tomato responds to P starvation with dramatic changes in gene expression resulting in the remodeling of many processes aimed at improving uptake and recycling of P as well as processes aimed at acclimation of plant growth and development by altering hormone signaling. We demonstrate that in many of these responses, SLs play a critical role.

## Results and discussion

### P starvation induces transcriptional reprogramming in tomato

**Fig. 1:**
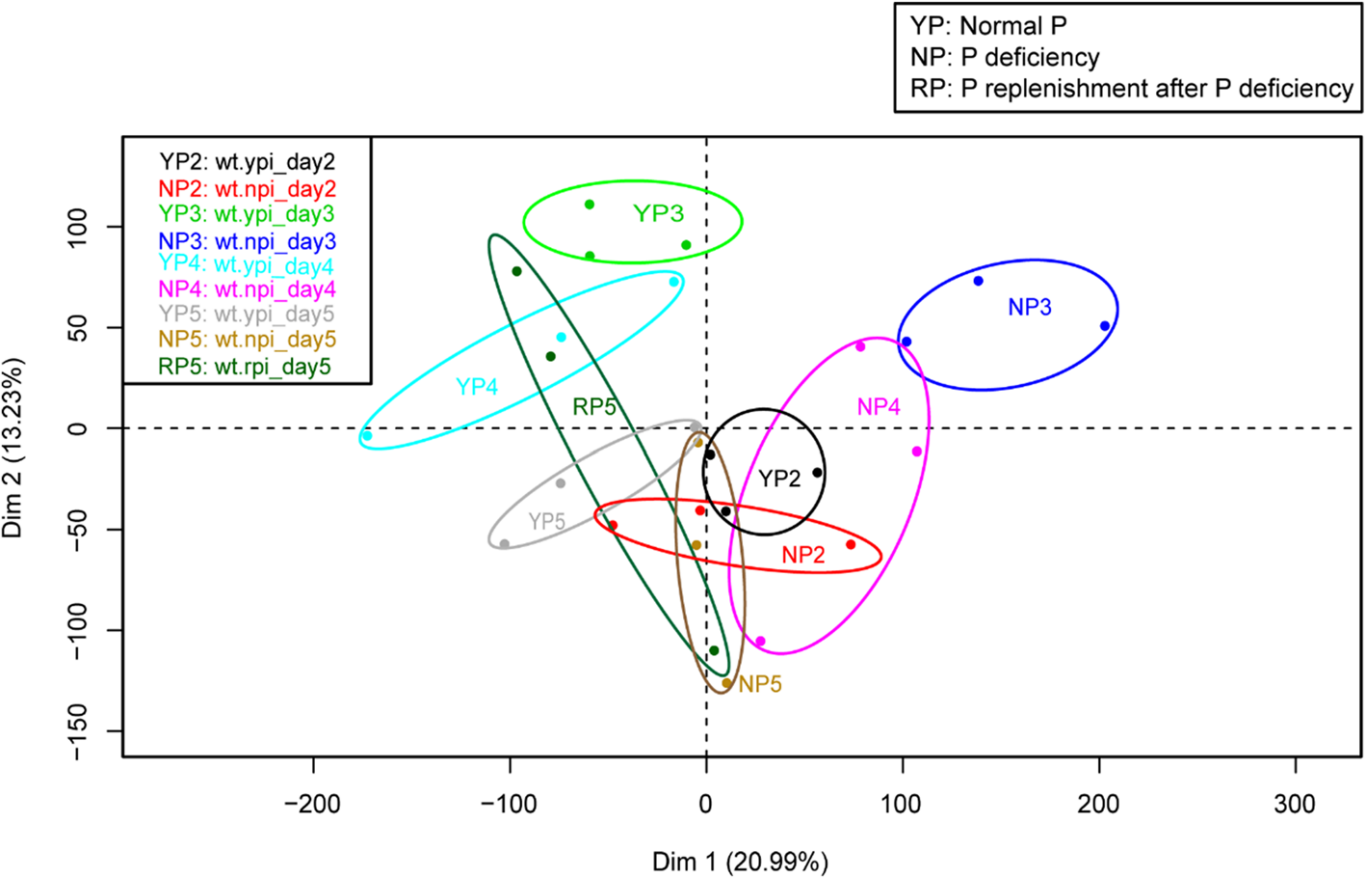
PCA of tomato root transcript profiles using RPKM. YP2, YP3, YP4 and YP5 represent 2, 3, 4 and 5 days of control P treatment, respectively. NP2, NP3, NP4 and NP5 represent 2, 3, 4 and 5 days of P starvation, respectively. RP5 represents one day of P replenishment after 4 days of P starvation

To visualize the global expression changes under P starvation over time and between treatments, principal component analysis (PCA) was performed (Fig. 1). The two major components explained 33% of the variation in gene expression between the samples. The biggest effect of P starvation on gene expression was observed at 3 and 4 days when the difference between NP3 and NP4 and their controls (YP3 and YP4) was the largest. The overall gene expression profile of the one-day P replenishment treatment (RP5) changed compared with NP4 to become more similar to the P sufficient treatments (YP3, YP4 and YP5) than the corresponding P starvation treatments (NP3, NP4 and NP5). This suggests a prompt phosphate stress recovery.

**Fig. 2:**
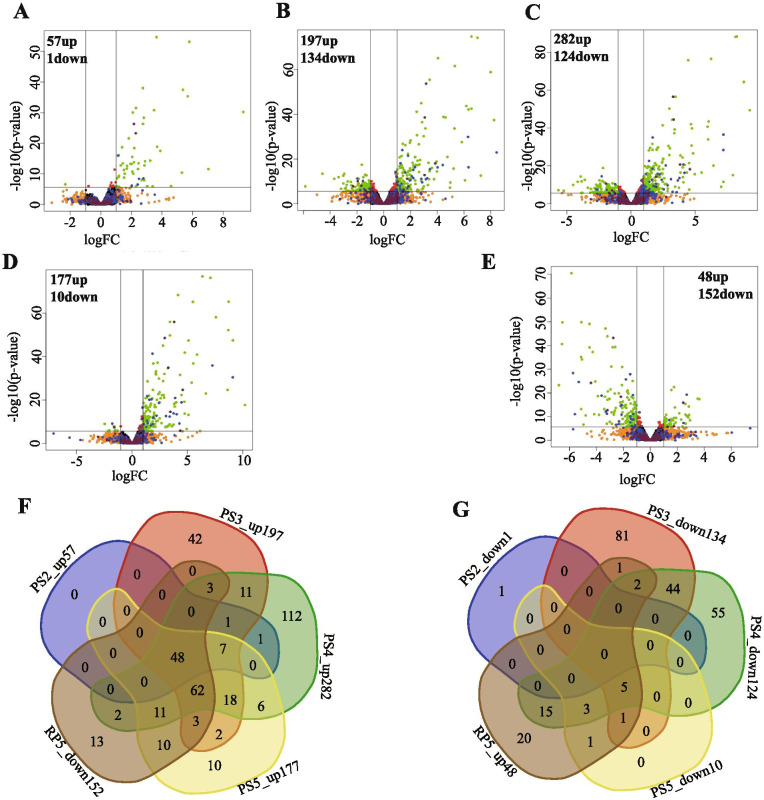
Volcano plots and Venn diagrams showing differentially expressed genes (DEGs) between P starvation and replenishment and their respective controls, at different time points. **A**-**D**, volcano plots of 2-day (NP2 vs YP2), 3-day (NP3 vs YP3), 4-day (NP4 vs YP4) and 5-day (NP5 vs YP5) P starvation. **E**, volcano plot of one-day P replenishment (RP5 vs NP5). **A**-**E**, red, green, orange and black indicate the position in the volcano plot, which itself indicates whether they pass or not the significance thresholds: P <  = 0.05 and log2FC >  = 1. Black indicates genes that did not pass any of the thresholds. Orange indicates genes that only pass the log2FC threshold. Red indicates genes that only pass the P threshold. Green indicates genes that pass both thresholds. Blue indicates genes containing plantiSMASH-predicted ‘biosynthetic’ domain [30]. Wine/purple genes belong to plantiSMASH-predicted biosynthetic gene clusters [30]. **F**-**G**, Venn diagrams of up- and downregulated DEGs, respectively, at different time points of P deficiency and down- and upregulated, respectively, by P replenishment. PS2_up, PS3_up, PS4_up and PS5_up represent upregulated genes of comparison NP2 vs YP2, NP3 vs YP3, NP4 vs YP4 and NP5 vs YP5, respectively. RP5_down represents the downregulated DEGs by P replenishment (RP5 vs NP5); PS2_down, PS3_down, PS4_down and PS5_down represent downregulated genes of comparison NP2 vs YP2, NP3 vs YP3, NP4 vs YP4 and NP5 vs YP5, respectively; RP5_up represents the upregulated DEGs by P replenishment (RP5 vs NP5)

Next, we investigated which genes show a large response to P starvation. For this purpose, we displayed the up- and down-regulated genes with fold change (FC) ≥ 2 (P ≤ 0.05) using a volcano plot. As shown in Fig. 2A, 2 days of P starvation resulted in 57 up-regulated genes and only 1 down-regulated gene compared to the control (YP2). Upon extending the P starvation treatment to 3 and 4 days (NP3 and NP4), the total number of DEGs increased to 331 and 406, respectively (Fig. 2B, C). Of these, 197 and 282 genes were upregulated, while 134 and 124 genes were down-regulated, respectively, compared with their respective controls. Interestingly, instead of a continuous increase, the number of differentially expressed genes (DEGs) in 5-day P starved samples (NP5) decreased to 187, of which 177 were up- and 10 downregulated (Fig. 2D, F, G). Apparently, the P starvation response reached a maximum at 4 days, possibly as a result of P shortage acclimation. These results are reflected in our PCA analysis, in which the total gene expression profile under P starvation on day 5 is more similar to the control than on day 3 and 4 (Fig. 1). The numbers of DEGs detected are highly comparable to those in maize, where 3 days of P starvation resulted in the differential expression (fold-change ≥ 2) of 283 genes (199 genes up; 84 down) [27]. In white lupin, however, after 9 days of P starvation 904 genes were differentially expressed (535 up; 396 down with FC ≥ 2, P ≤ 0.05) [28], while in *Brachypodium distachyon*, a 7-day low P treatment resulted in 1740 DEGs (1175 upregulated; 565 downregulated with FDR ≤ 0.01) [29]. The different numbers of DEGs upon P starvation in different studies probably depend on the different initial conditions, differences in the length of the P starvation period, the developmental stage used, and/or differences in the P starvation response between plant species.

Upon just 1 day of P replenishment, a virtually opposite picture compared to Fig. 2D appeared with 152 genes down- and 48 upregulated compared with the P starvation control (NP5) (Fig. 2E). Just one day of P replenishment reduced the expression of 139 P-starvation upregulated genes, and increased the expression of 28 genes that were repressed by P-starvation, indicating that these genes are highly sensitive to P availability (Fig. 2F, G).

Of the P starvation up-regulated genes across all time points, 48 DEGs (Suppl. Table 1) constitute the core P response genes, as they were shared between all P starvation time points and were down-regulated by P replenishment (Fig. 2F). Some of these genes are among the top most induced PSRs (Suppl. Table 1) and will be discussed below. To learn more about these 48 core P response genes, we performed Gene Ontology (GO) enrichment analysis (Suppl. Table 1). The 48 DEGs were enriched for biological processes such as ‘lipid metabolic process’, ‘glycolipid biosynthetic process’, ‘galactolipid biosynthetic process’, ‘liposaccharide metabolic process’ and ‘phosphate ion homeostasis’. The 48 DEGs were also enriched for molecular function (‘phosphatase activity’, ‘hydrolase activity’, and ‘phosphoric ester hydrolase activity’) and cellular component GO terms (Suppl. Table 1). These GO enrichment results are consistent with studies in barley, rice and *Arabidopsis* that showed that the majority of the DEGs in the root induced by P starvation and repressed by P replenishment are involved in P metabolism including phospholipid degradation, hydrolysis of phosphoric esters, sucrose synthesis, phosphorylation/dephosphorylation and post-transcriptional regulation [31–34].

There were no core P starvation down-regulated/P replenishment up-regulated genes common for all time points (Fig. 2G). Therefore, we performed GO enrichment analysis on the entire set of DEGs downregulated by P starvation and/or upregulated by 1-day P replenishment at any of the time points (Suppl. data set 1-7). No significantly enriched GO terms could be retrieved for the down-regulated DEGs at 2 and 5 days of P starvation (Suppl. Table 2). Four GO terms (‘response to abiotic stimulus’, ‘response to stimulus’, ‘response to heat’ and ‘response to inorganic substance’) were enriched in the 3- and 4-day P starvation down-regulated DEGs. For 3- and 4-day P starvation and RP5, GO enrichment analysis of downregulated DEGs showed enrichment for the biological process ‘response to stress’. Interestingly, the GO term ‘defense response’ is enriched in the 4-day P starvation downregulated as well as 5-day P replenishment upregulated DEGs. This GO category is composed of disease resistance and *DEFENSIN D1*-like genes, suggesting that the immune system was repressed to enable enhanced interaction with (beneficial) micro-organisms such as AMF, as reported previously [35]. Another 16 GO terms such as ‘response to nitrogen compound’, ‘nucleobase-containing compound biosynthetic process’ and ‘organic cyclic compound biosynthetic process’ are specific for 3-day P starvation downregulated DEGs (Suppl. Table 2), in accordance with results in rice roots for 22-day P starvation (enrichment of ‘nitrogen compound metabolic process’ and ‘N metabolism’ in down-regulated DEGs) [33, 36]. A link between P and N responses in plants has been suggested before. Overexpression of the P transporter gene, *OsPHT2*, enhanced N fixation in transgenic soybean under P deficiency [37]. Moreover, some gene expression responses to P and N starvation are similar and N availability controls the PSR in many plant species, such as *Arabidopsis*, wheat and rice [37–39]. Also the GO term ‘response to cadmium ion’ was enriched in the 4-day P starvation downregulated DEGs (Suppl. Table 2) and this was also observed in *A. thaliana* under P starvation [40].

Many of the P responsive genes are enriched for GO terms related to primary and secondary metabolism (Suppl. Table 1, Suppl. Table 2). This prompted us to create a more global overview of tomato plant metabolism under P deficiency using KEGG analysis. This showed that P starvation-induced and P replenishment-repressed DEGs are enriched in ‘lipid and carbohydrate metabolism’, ‘biosynthesis of other secondary metabolites, terpenoids’ and ‘polyketides metabolism’, ‘glycan biosynthesis and metabolism’ and ‘amino acid metabolism’ (Suppl. Figure 2A). Similar results were observed in *B. distachyon* and white lupin, with P starvation-induced DEGs enriched for lipid, carbohydrate and amino acid metabolism [28, 29]. Surprisingly, KEGG analysis on P starvation-repressed and P replenishment-induced DEGs showed that both are involved in lipid, carbohydrate and amino acid metabolism (Suppl. Figure 2B), suggesting that both up- and downregulation, probably of specific pathway branches, are involved in remodeling of lipid, carbohydrate and amino acid metabolism upon P starvation.

### SL biosynthesis and signaling is influenced by P starvation

The biosynthesis of the plant hormone SL has also been shown to be upregulated by P starvation [21, 41–43]. To study what the contribution is of SLs in the response to P, we assessed the expression of SL biosynthetic and signaling genes under the influence of P deficiency and P replenishment (Suppl. Figure 3A). To assess how SL biosynthetic and signaling are regulated by SL itself we looked at gene expression changes in a *CCD8* RNAi line (Suppl Fig. 3A). The SL biosynthetic genes *DWARF 27* (*D27*), *CCD8, MORE AXILLARY GROWTH 1* (*MAX1*) and *CYP722C* were all upregulated at any time point of P starvation (or from 3 days) and repressed again upon P replenishment, although mostly not significant. Unexpectedly, *CCD7* did not mirror this pattern, although at 5-day P starvation it was also upregulated compared with the control. Validation of these results using Reverse-Transcriptase quantitative PCR (RT-qPCR) for *D27* and *CCD8* showed the same pattern, confirming a significant up-regulation for most time points of P starvation and down-regulation upon P replenishment (Suppl. Figure 3B, C). In the *CCD8* RNAi mutant, expression of *D27*, *MAX1* and *CYP722C* were upregulated by P starvation, while *CCD7* was downregulated. The up-regulation of *D27* in the *CCD8* RNAi line was confirmed using RT-qPCR (Suppl Fig. 3C). Correlation analysis of RT-qPCR and RNAseq data showed a highly significant positive correlation (R^2^ = 0.9761; *P* < 0.01) (Suppl Fig. 3E) validating our RNAseq data. These results suggest that SL biosynthetic genes are all regulated by phosphate, but that SLs play an opposite role in regulating *D27*, *MAX1* and *CYP722C* on the one hand and *CCD7* on the other, under P starvation.

In contrast to the biosynthetic genes, the two *DWARF 14* (*D14*) homologs in tomato, encoding the SL receptor, were repressed by P starvation (from 3 days or 4 days of P starvation), and upregulated again by P replenishment (Suppl. Figure 3A), while in a study of *Arabidopsis*, *D14* was slightly upregulated by P deficiency in roots [16]. One of the two *MAX2* gene copies showed a similar pattern as D14 homolog 1 (Suppl. Figure 3A). *SUPPRESSOR OF MAX2 1-LIKE 6* (*SMXL6*) was slightly downregulated by P starvation at any of the time points and upregulated by one-day P replenishment (Suppl. Figure 3A). Except for 2-day P starvation, the expression pattern of *SMXL 8* was the opposite of that of *SMXL6* (Suppl. Figure 3A). Interestingly, the almost complete lack of SL biosynthesis in the mutant resulted in a strong upregulation of the SL receptor, *D14*, although *MAX2* expression was not affected (Suppl. Figure 3A). The expression of *SMXL6* and *SMXL8* were both slightly downregulated in the *CCD8* RNAi mutant (Suppl. Figure 3A).

### SLs influence the P-starvation induced transcriptional changes

**Fig. 3:**
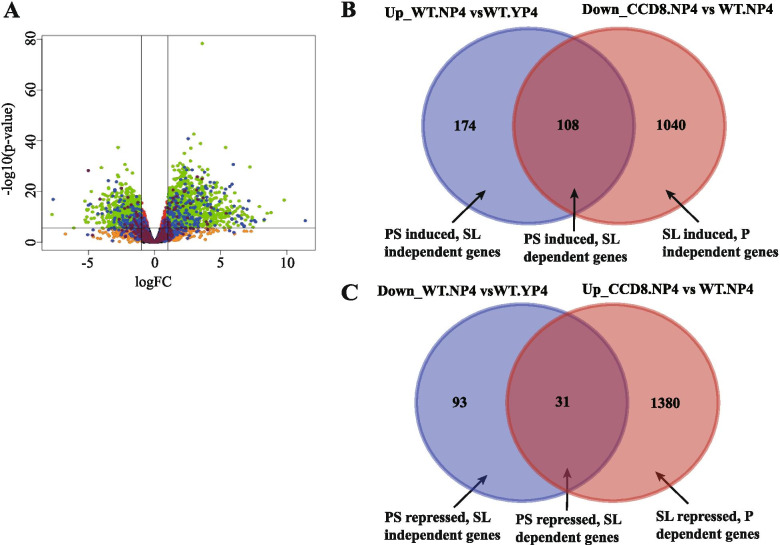
Venn diagrams and volcano plot illustrating DEGs in the *CCD8* RNAi line compared with WT tomato. **A**, volcano plot showing transcript differences between the *CCD8* RNAi line and WT tomato at 4-day P starvation. **B-C**, Venn diagrams showing the number of DEGs induced or repressed, respectively, in WT (compared with control conditions) and down regulated in the *CCD8* RNAi line (compared with WT) at 4-day P starvation

Above, we showed that SLs feedback on their own biosynthesis and signaling. Next, we compared gene expression changes under 4-day P starvation (strongest PSR) in the *CCD8* RNAi line and WT to verify if other P starvation DEGs also depend on SLs (Fig. 1, 2). Intriguingly, a large number of genes were significantly differentially expressed (compared with WT) in the roots of the *CCD8* RNAi mutant under P starvation with 1411 and 1148 DEGs up- and downregulated, respectively (Fig. 3A).

To find out of which P starvation response genes the changes in expression depend on the presence of SLs, we made Venn diagrams of the DEGs that are induced, respectively, repressed by 4-day P starvation in WT and are down-, respectively, upregulated in the *CCD8* RNAi line compared to WT (Fig. 3B, C). These comparisons split the DEGs into two sets with 108 and 31 genes that are only induced or suppressed, respectively, by P starvation in the presence of SL. In addition, these two Venn diagrams reveal the genes that are SL induced (1040 genes, Fig. 3B) and SL repressed (1380 genes, Fig. 3C), independent of P starvation. Both effects of SLs are also illustrated in a PCA (Suppl. Figure 4), which shows a large shift in gene expression in the RNAi line along PC1 (effect of the loss of SL) and a much smaller shift along PC2 in the RNAi line than in WT, illustrating the smaller change in gene expression as a result of P starvation in the absence of SL.

**Fig. 4:**
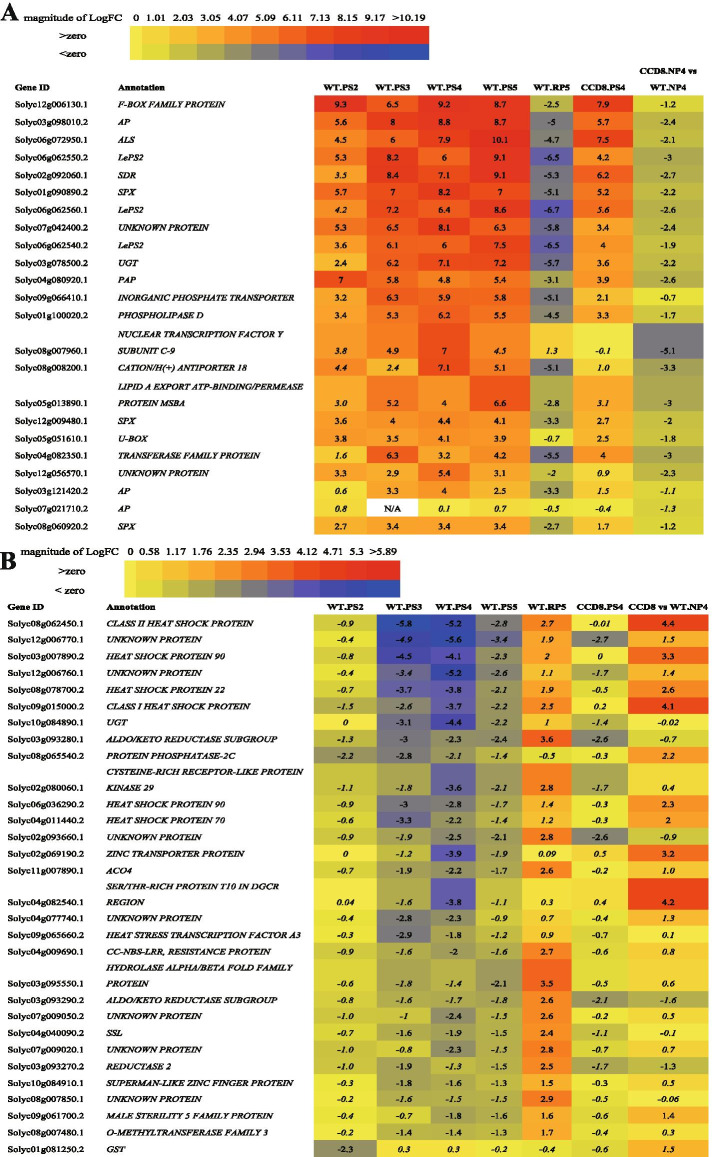
Heatmaps showing a selection of strongest induced and repressed DEGs in the roots of WT tomato and CCD8 RNAi line at different P starvation treatment times. **A**, the log2FC of the top 10 strongest P starvation-induced DEGS (at 2, 3, 4 and 5 days of P starvation) and their repression by P replenishment in WT, and DEGs at 4 days of P starvation in CCD8 RNAi line. **B**, the log2FC of the top 10 most repressed P starvation-induced DEGs (at 2, 3, 4 and 5 days of P starvation) and their upregulation by P replenishment in WT and CCD8 RNAi line. The heatmaps show expression profiles of the top 10 most induced/repressed P starvation DEGs; Values (in A and B) in italics represent the adjusted *P*-value < 0.05

To further analyze the role of SLs in the PSR, we assembled a rank list of the most strongly P-starvation-responsive genes in WT and the CCD8 RNAi line (Fig. 4A). Of the P starvation response genes in WT, three *LePS2* homologs (Solyc06g062540.2, Solyc06g062550.2, Solyc06g062560.1), one *NORGANIC PHOSPHATE TRANSPORTER* (Solyc09g066410.1) and the two *SPX* (Solyc01g090890.2, Solyc12g009480.1) have also been reported by others to be induced under P starvation in tomato [13, 26, 44, 45]. RT-qPCR confirmed that one of the *LePS2s* indeed displayed a strong induction in all time-point of P starvation (Suppl. Figure 3D). Our rank list also contains genes that have been reported as the strongest induced genes in rice under long-term P starvation (21-day) such as *INORGANIC P TRANSPORTER*, *PAP*, *AP* and *ABC TRANSPORTER-LIKE* (*ALS*) [33]. The latter authors also reported induced expression of an *SPX* that plays an essential role in P signaling for maintaining P homeostasis in plants [46]. *PAP*s are members of the most important class of *AP*s and play a crucial role in intra- and extracellular P scavenging and recycling under P deficiency [29, 47]. Secretion of AP or PAP by plants from the roots is an adaptive response to P stress to access bound P in the rhizosphere [48]. High in our rank list is an F-box family protein; this may be a negative regulator of the P starvation response just as the F-box homolog reported in *Arabidopsis* [49]. The Lipid A export ATP-binding/permease protein, MsbA, and Phospholipase D are two enzymes involved in remodeling of lipid metabolism, which is one of the adaptive mechanisms of plants to cope with P starvation as is the induction of the expression of P transporters, such as the *INORGANIC PHOSPHATE TRANSPORTER* (Fig. 4) [5]. In the roots of *B*. *distachyon*, the expression of several *SPX*, *PAP* and *P transporters* was also significantly induced by P starvation [29]. All the top-ranked P starvation-induced genes were effectively repressed upon one day of P replenishment (RP5) (Fig. 4A).

All these top-ranked P starvation induced DEGs (Fig. 4A), except *F-BOX FAMILY PROTEIN*, *ALS*, *INORGANIC P TRANSPORTER* and *U-BOX* are among the 108 DEGs (SL-dependent upregulation under P starvation) (Figs. 3B, 4A). The heat map shows that the induction of expression of the majority of these top responsive genes is much lower in the *CCD8* RNAi line than in WT (Fig. 4A, Suppl. Figure 5). In addition, some genes do not respond at all to P starvation in the *CCD8* RNAi line, like *NUCLEAR TRANSCRIPTION FACTOR Y SUBUNIT C-9*, 2 *CYTOCHROME P450*s (*P450s*), *2-OXOGLUTARATE-DEPENDENT DIOXYGENASE (2-ODD)*, *1-AMINOCYCLOPROPANE-1-CARBOXYLATE OXIDASE 6*, *AMP-DEPENDENT SYNTHASE AND LIGASE*, etc. (Suppl. Table 3) [50].

The above results show that many of the top-ranked PSR genes (Fig. 4A) found in the present study have also been reported in the literature and that for many of these, the response to P starvation depends on SLs. In *Arabidopsis* and rice, the root architecture response to low P was attenuated in SL biosynthesis mutants. Moreover, in *Arabidopsis*, rice and tomato, induction of the expression of several PSR genes by low P was compromised in SL mutants and could be complemented by the application of the synthetic SL, GR24 [18, 19, 51]. Here we show that SLs play an even more comprehensive role in the regulation of the P starvation response by controlling the expression of many PSR genes.

Among the top 10 strongest P-starvation-repressed DEGs (Fig. 4B) are a *GLUTATHIONE-S-TRANSFERASE* (*GST)*), several heat-shock/heat-stress related proteins, a *UDP-GLUCOSYLTRANSFERASE* (*UGT*) and several unknown proteins (Fig. 4B). In maize, a *UGT* and *GST* also displayed reduced expression in the roots in response to low P stress [27]. In addition, several other genes were repressed by P starvation and induced by P replenishment, making them interesting P response genes, such as *CYSTEINE-RICH RECEPTOR-LIKE PROTEIN KINASE*, *ALDO/KETO REDUCTASE*, *1-AMINOCYCLOPROPANE-1-CARBOXYLIC ACID* *(ACC) OXIDASE 4,* *STRICTOSIDINE SYNTHASE-LIKE* (*SSL*), *O-METHYLTRANSFERASE FAMILY 3* and *SUPERMAN-LIKE ZINC FINGER PROTEIN* (Fig. 4B). This is consistent with research in soybean showing that an *ALDO/KETO REDUCTASE* was differentially expressed in the shoot after 12 h P starvation [52]. In the roots of maize seedlings, a number of *S-ADENOSYLMETHIONINE-DEPENDENT METHYLTRANSFERASES* responded differentially to 3-day P starvation [27]. SSL could be an enzyme involved in the biosynthesis of alkaloids, possibly indicating that alkaloid biosynthesis is repressed under P starvation, which would fit with the repression of ‘defense response’ discussed above. The heat map shows that the repression of the expression of the majority of these top responsive genes is weaker in the *CCD8* RNAi line than in WT (Fig. 4B). Of the 31 DEGs identified in Fig. 3B (SL-dependent downregulation under P starvation), especially the down-regulation of *ZINC TRANSPORTER*, several *HEAT SHOCK PROTEINs*, *SER/THR-RICH PROTEIN T10 IN DGCR REGION* and *MALE STERILITY 5 FAMILY PROTEIN* are completely dependent on the presence of SLs (Fig. 4B, Suppl. Table 3).

### Lipid, phenylpropanoid and carotenoid biosynthesis are reprogrammed under phosphate starvation and this partially depends on strigolactones

**Fig. 5:**
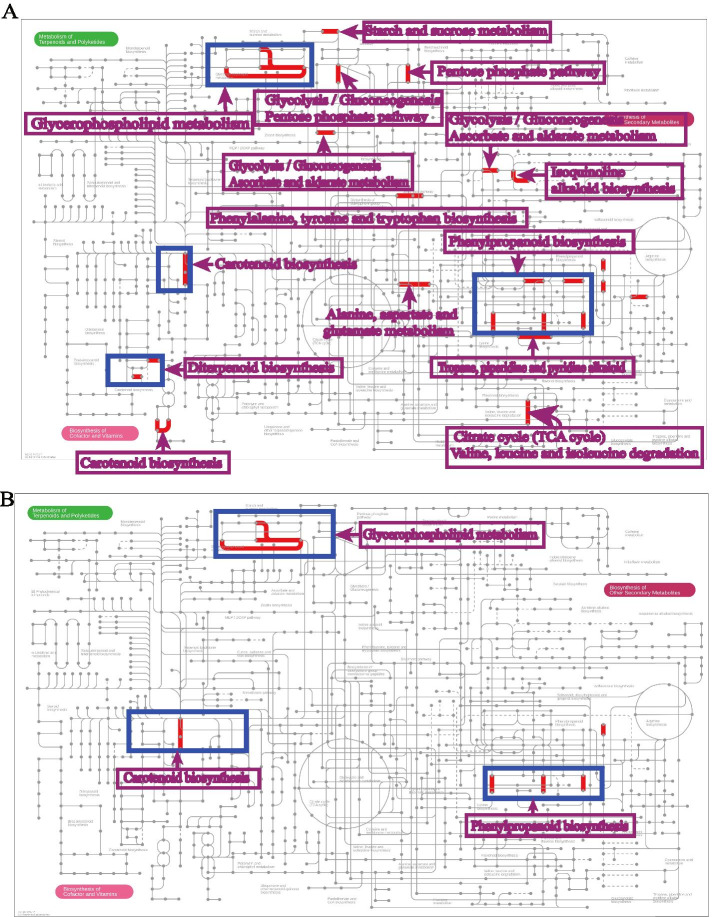
Visualization of Pstarvation-induced and SL-dependent changes in the expression of genes involved in secondary metabolism with iPath 3.0 [53, 54]. **A**, secondary metabolite biosynthesis and P starvation-induced DEGs (4-day) in WT. **B**, secondary metabolite biosynthesis and P starvation-induced and SL-dependent DEGs

Intriguingly, among the above-mentioned 108 DEGs, there were many metabolism-related genes, such as *P450*s, *2-ODD*, *UGT*, *GST*, *PHYTOENE SYNTHASE 3* and *CCD1-like* (Suppl. Table 3). This suggests that SLs are also involved in the regulation of metabolic reprogramming upon P starvation. To gain more detailed insight into the effect of phosphate starvation on metabolism and the role of SLs in this, we performed KEGG analysis and visualization using iPath3.0 with 282 genes (P starvation-induced genes in WT), 124 genes (P starvation repressed genes in WT), 108 genes (SL-dependent P starvation-induced genes) and 31 genes (SL-dependent P starvation downregulated genes) (Fig. 5, Suppl. Figure 6). For KEGG analysis, 129 (45.7%) out of the 282 genes, 43 (34.7%) out of the 124 genes, 18 (58.1%) out of the 31 genes and 44 (40.7%) out of the 108 genes could be annotated using BlastKOALA [55] (Suppl. Table 4-7). Upon P starvation, glycerophospholipid metabolism, and biosynthesis of carotenoids, diterpenoids and phenylpropanoids were induced (Fig. 5A, Suppl. Table 4), which (partially) required the presence of SLs (Fig. 5B, Suppl. Table 7). Even though the P starvation repressed gene set included more genes than the P starvation repressed/SL dependent gene set (Fig. 3C, Suppl. Table 5), the visual output of KEGG analysis of both sets looks similar (Suppl. Figure 6A, B), probably due to the limited annotation resolution of the KEGG pathway. The biosynthesis of pantothenate and CoA, lysine and alkaloids are repressed by P starvation, and this is clearly SL-dependent (Suppl. Figure 6A, B, Suppl. Table 5, 6).

**Fig. 6:**
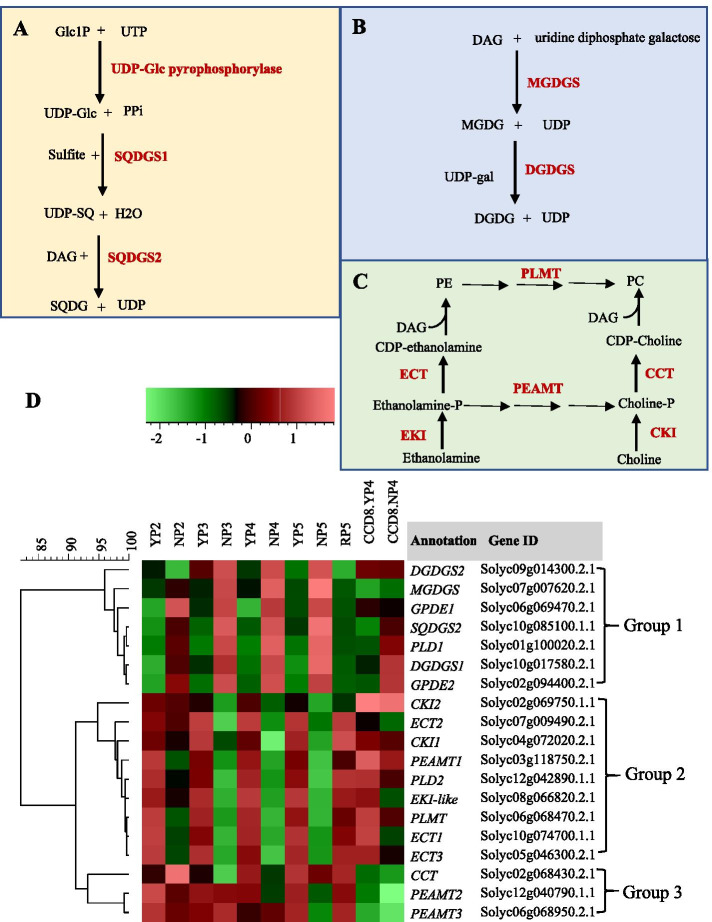
Expression profiles of lipid metabolism related genes in the roots of WT tomato and *CCD8* RNAi line under control P and P starvation treatment. A, schematic representation of SQDG biosynthesis [57]. **B**, schematic representation of DGDG biosynthesis. **C**, schematic representation of phosphatidylcholine (PC) biosynthesis [58]. **D**, hierarchical clustering diagram of lipid metabolism related genes in WT and *CCD8* RNAi line at 4 days of normal P and P starvation. YP4 and NP4 represent tomato WT at 4 days of control P and P starvation, respectively. CCD8.YP4 and CCD8.NP4 represent the *CCD8* RNAi line at 4 days of control P and P starvation, respectively. Glc1P, Glucose 1-phosphate; UTP, uridine-5′-triphosphate; PPi, pyrophosphate; SQ, sulfoquinovose; SQDG, sulfoquinovosyldiacylglycerol; DAG, 1,2-diacylglycerol; MGDG, monogalactosyldiacylglycerol; DGDG, digalactosyldiacylglycerol; PE, phosphatidylethanolamine; CDP, cytidine diphosphate; PC, Phosphatidylcholine; *DGDGS*, *DIGALACTOSYLDIACYLGLYCEROL SYNTHASE*; *AGPL*, *ADP-GLUCOSE PYROPHOSPHORYLASE*; *PFK*, *PHOSPHOFRUCTOKINASE*; *GPDE*, *GLYCEROPHOSPHODIESTERASE*; *MGDGS*, *MONOGALACTOSYLDIACYLGLYCEROL SYNTHASE*; *SQDGS*, *SULFOQUINOVOSYLDIACYLGLYCEROL SYNTHASE*; *PLD*, *PHOSPHOLIPID DEGRADATION*; *PEAMT, PHOSPHORYLETHANOLAMINE N-METHYLTRANSFERASE*; *CCT*, *CTP-PHOSPHOCHOLINE CYTIDYLYLTRANSFERASE*; *EKI*, *ETHANOLAMINE KINASE*; *ECT*, *CTP-PHOSPHOETHANOLAMINE CYTIDYLYLTRANSFERASE*; *GPAT*, *GLYCEROL-PHOSPHATE ACYLTRANSFERASE*; *CKI*, *CHOLINE KINASE*; *PLMT*, *PHOSPHOLIPID N-METHYLTRANSFERASE*; *GT8*, *GLYCOSYLTRANSFERASE FAMILY 8*; *SPS*, *SUCROSE PHOSPHATE SYNTHESIS*; *PGM*, *PHOSPHOGLYCERATE MUTASE*; *PEAMT*, *PHOSPHOETHANOLAMINE N-METHYLTRANSFERASE*

The above result suggesting that the remodeling of phospholipid metabolism under P starvation is SL-dependent prompted us to look in more detail into lipid metabolism. Intriguingly, hierarchical clustering analysis (HCA) of lipid metabolism related genes shows that several groups of genes can be distinguished, with one group being upregulated under P starvation, one being downregulated and one that does not respond to P starvation (Fig. 6). Genes in Group 1 are induced by P starvation and repressed by P replenishment; they mainly represent genes involved in galactolipid biosynthesis, sulfolipid biosynthesis and phospholipid degradation such as *MONOGALACTOSYLDIACYLGLYCEROL SYNTHASE* (*MGDGS*, Solyc07g007620.2.1), *DIGALACTOSYLDIACYLGLYCEROL SYNTHASE* (*DGDGS1*, Solyc10g017580.2.1; *DGDGS2*, Solyc09g014300.2.1) and *SULFOQUINOVOSYLDIACYLGLYCEROL SYNTHASE* (SQDGS2, Solyc10g085100.1.1) (Fig. 6A, B, D). Intriguingly, the genes involved in phospholipid degradation like *PHOSPHOLIPASE D 1* (*PLD1*, Solyc01g100020.2.1) and *GLYCEROPHOSPHODIESTERASE* (*GPDE1*, Solyc06g069470.2.1, Solyc02g094400.2.1) are also present in Group 1 (Fig. 6D). Overall, under P deficiency, plants reduce the demand for P in lipids by substituting P-free galactolipids, such as DGDGs, and sulfolipids, such as SQDGs, for phospholipids [5]. Indeed, an increased concentration of galactolipids and sulfolipids was reported in the leaves of *Arabidopsis* under P deficiency [56] and the expression of *SQDGS1* and *SQDGS2* is induced in seedlings of *Arabidopsis* and rice under P starvation [5]. In the *CCD8* RNAi line, however, the expression of some Group 1 genes was not induced, such as *DGDGS2*, *GPDE1* and *MGDGS* (Fig. 6D), showing that the remodeling of lipid biosynthesis as induced by P starvation is (partially) SL dependent. Group 2 represents genes with an opposite expression profile: they are repressed by P starvation (Fig. 6C, D). Genes in this group are mostly involved in phospholipid biosynthesis. Most of these genes are not or much less repressed by P starvation in the *CCD8* RNAi line. The downregulation of the expression of *PHOSPHORYLETHANOLAMINE N-METHYLTRANSFERASE* (*PEAMT*)*1*, *CHOLINE KINASE* (CKI) 1 and 2 in Group 2, for example, is completely absent in the *CCD8* RNAi line, showing that the down-regulation of phospholipid biosynthesis by phosphate shortage depends on SLs (Fig. 6D). *PEAMT 2*, 3 and *CTP-PHOSPHOCHOLINE CYTIDYLYLTRANSFERASE* (CCT) in Group 3 are not responding to P starvation but are highly repressed in the *CCD8* RNAi line (Fig. 6B). Taken together, our data show that a large part of the changes in lipid metabolism under P starvation depends on SLs, consistent with our KEGG analysis on the 108 P starvation induced SL-dependent DEGs.

### SLs affect other phosphate starvation related hormones

As discussed above, plant hormones play an important role in the acclimation of plants to P starvation. Using the same strategy as for other metabolic pathways, we also analyzed how the biosynthesis of other hormones under P starvation is affected by SLs.

#### Brassinosteroids

Brassinosteroids regulate plant growth and development [59] and have been linked to the SLs through BES1 (BRI1-EMS-SUPPRESSOR1), a positive regulator of brassinosteroid signaling, that is degraded as a result of SL signaling [60]. To visualize the interaction between P starvation, SLs and brassinosteroids, we performed HCA with the brassinosteroid biosynthetic pathway genes. Two clusters with different expression patterns showed up in the HCA (Suppl. Figure 7). Genes in Group 1 displayed a lower expression in the *CCD8* RNAi line than in WT, independent of P availability. In contrast, the genes in Group 2 exhibited a higher expression in the *CCD8* RNAi line, also independent of P availability. In Group 1 we find many of the *P450s* involved in the brassinosteroid pathway. In Group 2 there was one exception to the general pattern: the expression of *CYP90D2* depends on both SL and P availability. Similarly, expression of *CYP92A6* and *DELTA (24)-STEROL REDUCTASE HOMOLOG 2* (*DWF1_H2*) also depends on both SL and P availability. However, the expression of *C-4α–STEROL-METHYLOXIDASE2 HOMOLOG2* (*SMO2_H2*) in Group 2 was induced by P starvation in both WT and the *CCD8* RNAi line showing that *SMO2_H2* is P starvation responsive and SL independent. Similarly, *DELTA(7)-STEROL-C5(6)-DESATURASE HOMOLOG 1* (*STE_H1*) in Group 1 is downregulated by P starvation both in WT and the *CCD8* RNAi line. Overall, these results indicate that most of the biosynthetic genes of brassinosteroid and steroid biosynthesis do not respond to P starvation but strongly—positively or negatively—depending on the presence of SLs. BES1, the positive regulator of brassinosteroid signaling, is a substrate of MAX2 and therefore degradation of BES1 is promoted by SLs [60]. Our results suggest that the feedback relationship between SLs and brassinosteroids extends beyond signaling, also to brassinosteroid biosynthesis.

#### Ethylene and auxin

As described in the introduction, ethylene plays a role in the acclimation to P starvation by affecting morphological changes in the root system [61]. In P starved adventitious roots of maize, the release of ethylene, and the content of ACC and ACC oxidase (catalyzing a rate-limiting step in ethylene formation) all decreased under P deficiency [62]. Also in soybean, *ACC OXIDASE 1* expression decreased under P starvation [63, 64]. In the present study, *ACC OXIDASE 4* (Solyc11g007890.1) is consistently repressed by P starvation (at 3, 4 and 5 days) and upregulated by P replenishment (Fig. 4A). Interestingly, in the *CCD8* RNAi mutant, *ACC OXIDASE 4* is not downregulated under P starvation. Another ethylene-related gene, *ETHYLENE RESPONSIVE FACTOR REQUIRED FOR NODULATION* 3 (*ERN3-like*, Solyc01g091760.2) is induced at PS4 in WT (Suppl. Table 5). *ERN1* and *ERN2* act as transcriptional activators, while *ERN*3 acts as a putative repressor of ERN1/ERN2-dependent transcriptional activation in root hairs [65]. Possibly this ERN3 is responsible for P starvation induced changes in root hair elongation in tomato. In the *CCD8* RNAi line there was no significant change in *ERN* expression in response to P starvation (Suppl. Table 4). The same was observed for the *AUXIN RESPONSIVE PROTEIN* (SAUR-like protein, Solyc07g045060.1) (Suppl. Table 4), which is induced at PS4 in WT just as reported for soybean [63], but less in the *CCD8* RNAi line. Acclimation in root architecture under P starvation thus seems to require the repression of ethylene biosynthesis and activation of auxin signaling [66] and we show here that this (partially) depends on the presence of SLs.

#### Gibberellins

**Fig. 7:**
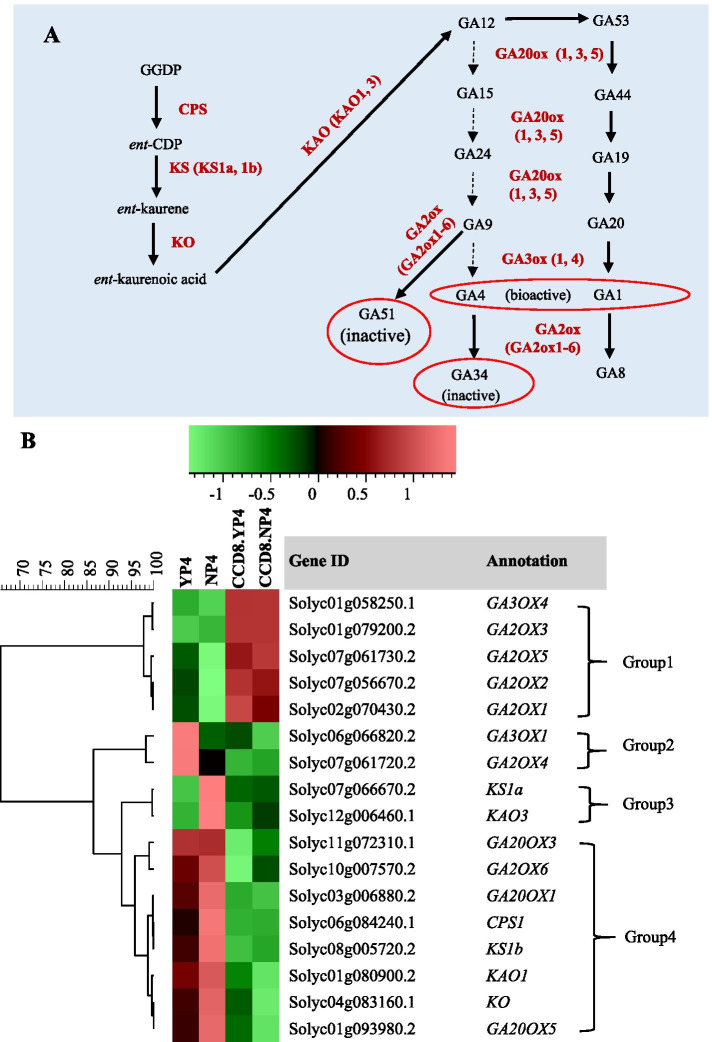
Expression of gibberellin pathway genes in the root of WT tomato and *CCD8* RNAi line under normal P and P starvation. **A**, schematic representation of the gibberellin pathway [67, 68]. **B**, hierarchical clustering diagram of gibberellin pathway related genes in WT and CCD8 RNAi line at 4 days of normal P and P starvation. YP4 and NP4 represent tomato WT at 4 days of control P and P starvation, respectively. CCD8.YP4 and CCD8.NP4 represent the *CCD8* RNAi line at 4 days of control P and P starvation, respectively. *GA3OX*, *GIBBERELLIN 3-OXIDASE*; *GA2OX*, *GIBBERELLIN 2-OXIDASE*; *KS*, *ENT-KAURENE SYNTHASE*; *KAO*, *ENT-KAURENOIC ACID OXIDASE*; *GA20OX*, *GIBBERELLIN 20-OXIDASE*; *CPS*, *COPALYL DIPHOSPHATE SYNTHASE; KO*, *ENT-KAURENE OXIDASE*

Based on HCA, tomato gibberellin biosynthesis genes cluster into 4 groups (Fig. 7B). The genes of Group1 are slightly repressed by P starvation and strongly repressed by SLs (higher expression in the *CCD8* RNAi line; Fig. 7). This cluster includes four gibberellin 2-oxidases (GA2OX1, 2, 3, 5) that convert GA9 to inactive forms such as GA51 and GA34 and one gibberellin 3-oxidase (GA3OX) that catalyzes the conversion of GA9 to the active GA1 and GA4. In contrast to Group 1, expression of genes in Group 4—which includes four genes responsible for GA biosynthesis (*GA20OX1*, *3*, *5* and *GA2OX6*) and four genes involved in the common precursor GA pathway—is much lower in the *CCD8* RNAi mutant compared with WT, so they are activated by SLs, and they are activated by P starvation in WT (Fig. 7). Interestingly, genes (*GA3OX1* and *GA2OX4*) in Group 2 are depending on SLs only when there is sufficient P. Under P starvation, these two genes had very low expression and this low expression does not depend on SL biosynthesis (Fig. 7). *KS1a* and* KAO3* in Group 3 are involved in the common precursor GA pathway; they were induced by P starvation in WT, and this depended on the presence of SLs, as they were not upregulated in the *CCD8* RNAi mutant (Fig. 7).

It has been reported that GA regulates the biosynthesis of SLs and there is crosstalk between GA and SL signaling [69, 70]. The present study uncovers another side of this cross-talk between SL and GA showing that SLs also regulate GA biosynthesis. Firstly, several *GA2OX* paralogs, which are involved in the inactivation of GA and clustered together in Group 1, were more expressed in the *CCD8* mutant. Thus, SL represses the expression of these genes that encode inactivating enzymes probably resulting in the presence of more active GA. Secondly, the expression of genes encoding the GA precursor pathway (Group 4) was lower in the *CCD8* RNAi line, which suggests that SL plays a positive role in upregulating these genes. Thirdly, GA3OX1 responsible for the production of active GA1 and/or GA4 is also upregulated by SL although its paralogue, *GA3OX4*, is repressed by SL regardless of P availability. This could imply that in the root different *GA3OX* paralogues are responsible for the production of different active GAs and/or only the biosynthesis of one active form of GA is induced by SL. P starvation in *Arabidopsis* results in elongation of lateral roots and inhibition of primary root growth [71]. The application of bioactive GAs to the shoot of *Arabidopsis* has been shown to promote primary root elongation and increase the number of lateral roots [72], showing that GA affects root architecture. In a report on the role of GA in the low P response in *Arabidopsis* seedlings grown on agar plates, Jiang et al. reported that the expression of the GA deactivating *GA2OX2* increases while expression of the biosynthetic/activating *GA20OX1* and *GA3OX1* decrease under low P conditions [2]. Our results only confirm the down regulation of *GA3OX1*. Jiang et al. also showed that the level of bioactive GA4 is downregulated under low P conditions, however, this was measured in entire seedlings, so including the shoot [2]. We show here the expression of many more genes (putatively) involved in GA metabolism. Especially the root-specific upregulation of the Group 3 and 4 genes—which include all early GA pathway genes—under P starvation supports the work on *Arabidopsis* [2]. Our results suggest that SLs play an essential role in the regulation of GA biosynthesis, repressing inactivation and upregulating biosynthesis, which possibly increases the level of active GA in the roots.

#### Abscisic acid

**Fig. 8:**
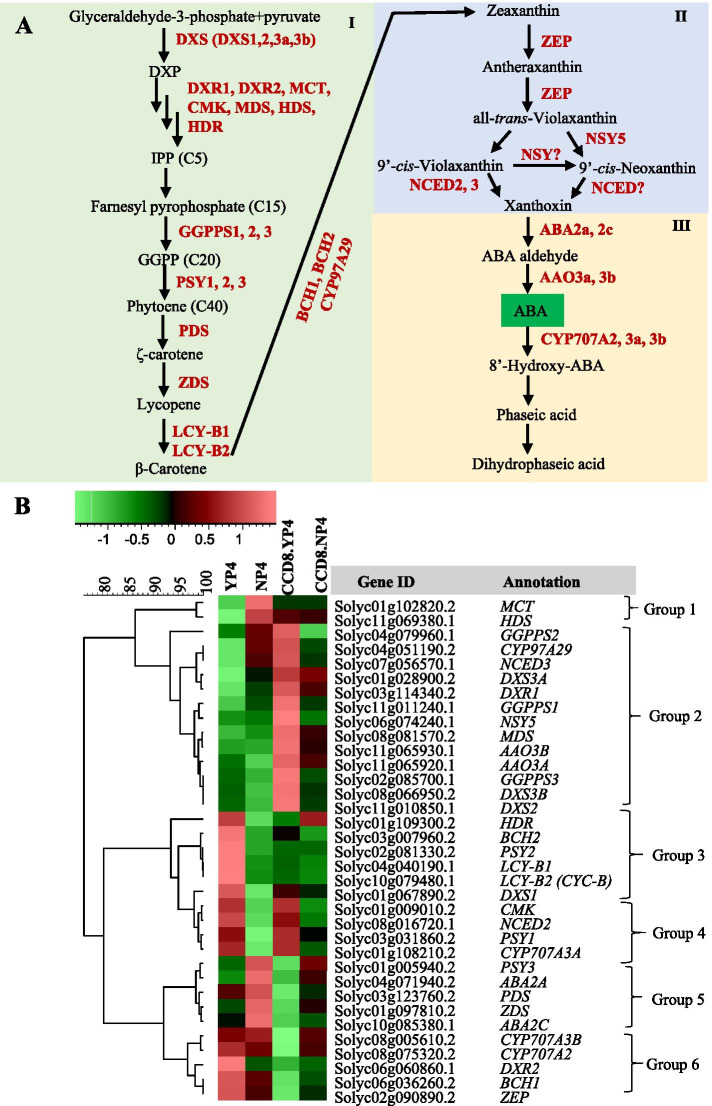
Expression profiles of ABA biosynthetic and catabolic genes in the root of WT tomato and *CCD8* RNAi line under normal P and P starvation. **A**, schematic representation of ABA biosynthesis and inactivation [82]: I represents carotenoid precursor biosynthesis; II represents the formation of epoxycarotenoids and their cleavage in the plastids, III represents reactions in the cytosol for the formation of ABA. **B**, hierarchical clustering diagram of ABA biosynthesis pathway related genes in WT at 4-day normal P and P starvation. YP4 and NP4 represent tomato WT at 4 days of control P and P starvation, respectively. CCD8.YP4 and CCD8.NP4 represent the *CCD8* RNAi line at 4 days of control P and P starvation, respectively. *DXS*, *1-DEOXY-D-XYLULOSE-5-PHOSPHATE SYNTHASE*; *DXR*, *1-DEOXY-D-XYLULOSE 5-PHOSPHATE REDUCTOISOMERASE*; *MCT*, *2-C-METHYL-D-ERYTHRITOL 4-PHOSPHATE CYTIDYLYLTRANSFERASE*; *CMK*, *4-DIPHOSPHOCYTIDYL-2-C-METHYL-D-ERYTHRITOL KINASE*; *MDS*, *2-C-METHYL-D-ERYTHRITOL 2,4-CYCLODIPHOSPHATE SYNTHASE*; *MDS*, *4-HYDROXY-3-METHYLBUT-2-EN-1-YL DIPHOSPHATE SYNTHASE*; *HDR*, *4-HYDROXY-3-METHYLBUT-2-ENYL DIPHOSPHATE REDUCTASE*; *GGPPS*, *GERANYLGERANYL PYROPHOSPHATE SYNTHETASE*; *PSY*, *PHYTOENE SYNTHASE*; *PDS*, *PHYTOENE DESATURASE*; *ZDS*, *ZETA-CAROTENE DESATURASE*; *LCY*, *LYCOPENE BETA CYCLASE*; *BCH*, *BETA-CAROTENE HYDROXYLASE*; *ZEP*, *ZEAXANTHIN EPOXIDASE*; *NSY*, *NEOXANTHIN SYNTHASE*; *NCED*, *EPOXYCAROTENOID DIOXYGENASE*; *AAO*, *ARABIDOPSIS ALDEHYDE OXIDASE*

SLs and ABA are both derived from the carotenoid pathway and both play a role in the response to P starvation. To visualize their interaction, genes involved in carotenoid and ABA biosynthesis were used for HCA, which revealed six clusters (Fig. 8). The most striking cluster is Group 2, consisting of genes of which the expression is strongly upregulated in the *CCD8* RNAi line, especially under normal P conditions. This cluster contains genes from all over the pathway but seems to be enriched in ABA biosynthesis-related genes, such as *CYP97A29*, *NCED3*, *NSY5*, *AAO3b* and *AAO3a* (Suppl. Figure 8). Possibly, other genes in this cluster, encoding earlier steps in the pathway, are dedicated (also) to ABA biosynthesis and therefore show the same expression pattern. Intriguingly, these genes are strongly negatively regulated by SL, independent of P presence. Genes involved in ABA catabolism show an opposite trend, at least to some extent. *CYP707A2* and *CYP707A3b* in Group 6 show the opposite pattern and are activated by SLs. This trend supports the reported negative relationship between SL and ABA in rice and lotus [50, 73–75] and suggests that the relationship is based on direct negative feedback from SL on ABA biosynthesis and positive feedback on ABA degradation, the latter of which is consistent with research by others [76, 77]. This response seems to differ between root and shoot since it has been suggested that there is no antagonism between SL and ABA in the shoot of tomato and other dicots [78]. Other striking patterns are formed by Groups 3 and 4, containing genes of which the expression is down-regulated by P starvation. In Group 3 the expression is not down-regulated in the CCD8 RNAi line, suggesting that this down-regulation is SL-dependent, while Group 4 down-regulation also occurs in the *CCD8* RNAi line, so is SL-independent. This last group also includes two ABA-related genes, NCED2 and CYP707A3a suggesting that they may be involved in a SL-independent pathway for the down regulation of ABA under P starvation. Genes clustering in Groups 1 and 5, finally, were induced by P starvation. In Group 1, this up-regulation did not occur in the *CCD8* RNAi line suggesting that the up-regulation is SL-dependent, while in Group 5 the up-regulation is more or less SL-independent. Interestingly, this latter group includes ABA biosynthesis related genes, suggesting that there is also P starvation-induced ABA biosynthesis that is not controlled by SL. The picture emerging from all this is still not clear; ABA regulation is partially dependent on SL and partially not, both under conditions of normal P as well as under P starvation. Others have reported on the crosstalk between ABA and SLs and their role in abiotic stress responses such as drought and P starvation [74, 75, 78, 79]. A recent study suggests that zaxinone, another apocarotenoid metabolite just as SL, in *Arabidopsis* acts as a stress signal that positively regulates both ABA and SLs, while in the mycorrhizal rice it is a negative regulator of SLs [80, 81].

### Conclusions and prospects

Here we show that, within days, tomato strongly responds to P starvation with dramatic changes in gene expression reaching the highest level of response after 4 days. This response can be almost completely negated by P replenishment. We show that the expression of genes involved in metabolism of the plant hormones ABA, GA, ethylene and auxin dramatically changes in response to P starvation, which to a considerable extent depends on SLs. We also identified massive changes in the expression of genes involved in lipid, phenylpropanoid and carotenoid biosynthesis under phosphate starvation, which to a large extent also depend on the presence of SLs. Together this shows that the role of SLs in the acclimation of plants to P stress goes beyond the already reported inhibition of shoot branching and adaptation of root architecture, and also involves other classic P starvation responses such as remodeling of lipid metabolism and changes in hormone homeostasis.

## Methods

### Plant materials and growth conditions

WT *Solanum lycopersicum* L. cv Craigella (LA3247) and a *SlCCD8* RNAi line (line 9) in the same background were used [83]. Tomato seeds were germinated and grown in hydroponics on half-strength Hoagland for 14d, after which treatments including continuous normal P [18], P deficiency by using half-strength Hoagland solution without P (NP) (omission of KH_2_PO_4_; osmotic potential and K^+^ concentration were kept constant by substituting KNO_3_ for KH_2_PO_4_), P replenishment after P deficiency (RP) (for details of the experimental design see Suppl. Figure 1). For the normal P and P deficiency treatments, WT plants were harvested after 2, 3, 4 and 5 days of treatment (normal P: YP2, YP3, YP4 and YP5; P deficiency: NP2, NP3, NP4 and NP5); the *SlCCD8* knock-down plants were harvested after 4 days for P deficiency treatment (CCD8_NP4) and control (CCD8_YP4); for the P replenishment treatment, WT plants were harvested after 4-day P deficiency and 1-day replenishment (RP5). Each treatment consisted of three biological replicates. Roots were immediately frozen in liquid nitrogen and stored at -80 °C until further use.

### Total RNA isolation and library preparation

Total RNA from root samples was extracted using the RNA sample preparation kit RNeasy Mini Kit (QIAGEN) combined with TRizol reagent (Invitrogen), and genomic DNA was digested using RNase-Free DNase (Qiagen, USA). RNA integrity was evaluated by 1.0 agarose gel electrophoresis. Total RNA was quantified with a Nanodrop 2000 (Thermo scientific, Wilmington, USA) and samples were used for RNAseq library construction only when the OD 260/280 was higher than 1.8 and the OD 260/230 higher than 2.0.

For RNAseq cDNA library synthesis, the Illumina RS-122–2103 TruSeq® Stranded mRNA HT kit was used. An Agilent 2100 Bioanalyzer was used for library quality checking. To analyse library concentration, a Pico green concentration measurement using Tecan was performed. An average of 30 libraries was multiplexed and loaded on each lane of the Illumina Hiseq flow cell. Sequencing was then performed on a Sanger/Illumina 1.9 with 50 bases single end run, according to the manufacturer’s instruction. Reads were filtered with the program Trimmomatic [84] to remove the adaptor sequences, empty reads, short reads (< 25 bp), reads with a N ratio greater than 10% and low-quality sequences. An overview of raw read numbers, and trimming and mapping statistics are provided in Suppl. Table 8.

### Data analysis

Trimmed reads were mapped to the *Solanum lycopersicum* reference genome version SL 2.50 ITAG2.40 (http://solgenomics.net/) with CLC Genomics Server 8.5.2. Read alignment and quantification were performed with CLC Genomics Server 8.5.2 using the default settings for RNA-Seq mapping and analysis, and using quantile normalization. A summary of trimming and mapping statistics is provided in Suppl. Table 8. Genes expression levels were calculated as reads per kilobase of transcript per million mapped reads (RPKM). We performed PCA with the expression of the genes (RPKM value) using the package FactoMineR in R. Differential expression analysis was carried out in R with the edgeR package [85]. DEGs between each control and treatment were identified using the following thresholds: P value ≤ 0.05 and ^2^log-transformed FC ≥ 1 or ≤ -1 (included in supplementary data set 1-8). FC and P values of all the genes under treatment conditions, compared with their control, were plotted in R with volcano plots. Venn diagrams (http://bioinformatics.psb.ugent.be/webtools/Venn/) were used to show the DEGs under different comparisons.

### Validation of SL biosynthetic and P starvation marker gene expression

Eight hundred ng of the above-described RNA samples was used to synthesize cDNA using RevertAid Reverse Transcriptase (Thermo Fisher Scientific). The final volume of cDNA was diluted into 100 μL for the RT-qPCR reaction. Ten microliters containing 1 μL cDNA template, 4 μL primers mixture (300 nM), 2 μL 5 × EvaGreen Mix and 3 μL *Milli*-*Q* water were used for one reaction. The RT-qPCR was performed using: stage 1: 50 °C 2 min; stage 2: 95 °C 10 min; stage 3: 95 °C 15 s, 60 °C 1 min, 45 cycles). Primers used for RT-qPCR are shown in Suppl. Table 9. To test the primers’ specificity, whether a clear curve was present in the dissociation analysis was checked. PCR efficiency was calculated using a dilution series of cDNA template. Tomato reference genes were used as described before [86]. The relative expression in the different treatments was normalized to the average expression level of two reference genes as listed in Suppl. Table 9. The expression of these two reference genes across treatments and samples was stable at a CT of 20.7 ± 0.45 and 20.5 ± 0.88 for reference gene SGN-U584254 and SGN-U563892, respectively.

### Hierarchical clustering analysis of metabolism related genes

The RPKM expression value of metabolism related genes of interest under different treatments were submitted to GeneMaths XT (https://www.applied-maths.com/genemaths-xt). The expression values were Log_2_-transformed and mean-centred. The normalized data were used to perform hierarchical clustering analysis. The pairwise distance was calculated by Euclidean distance (with variance) and summarized by the UPGMA (unweighted pair group method with arithmetic mean) method.

### Functional analysis and visualization

PlantRegMap (Plant Transcriptional Regulatory Map) [87–89] was used for GO enrichment analysis at a P-value of 0.01. The list of gene identifiers and log2FC values from each condition (P repletion, P starvation, P replenishment) were imported into the MapMan software version 3.5.1R2 [90], and assigned to functional categories [85] using the tomato mapping file ‘Slyc_ITAG2.3'. For KEGG analysis, KO identifiers of *Solanum lycopersicum* genes were obtained using BlastKOALA [55]. For the visualization of KEGG pathways, Interactive Pathways Explorer v3 (iPath3.0) was used [91].

## Supplementary Information

**Additional file 1:Suppl. Fig. 1**. Experimental design of the P starvation RNAseq experiment. **Suppl. Fig. 2**. KEGG pathway enrichment for induced (A) and repressed (B) DEGs in tomato roots upon P starvation and P replenishment. **Suppl. Fig. 3**. Heatmap showing fold change of SL related genes in RNAseq dataset and RT-qPCR validation of SL biosynthetic and P starvation marker genes. A, heatmap showing fold change of genes involved in the SL biosynthetic and signaling pathway under P starvation for different time periods and P replenishment in WT tomato and CCD8 RNAi line. A fold change in bold indicates significance (*P*<0.05). B-D, relative expression of *D27* (B), *CCD8* (C) and *LePS2* (D) upon P starvation (and P replenishment) (n = 3). The gene expression level in 2-day control wild‐type plants (YP2) was set to 1. Error bars represent standard error of the mean. **, 0.01>P; *, 0.01 < *P *< 0.05; NG, not significant. E, comparison of RT-qPCR and RNA-seq data. A Pearson correlation coefficient of 0.9761 (*P *< 0.01) is observed between the RNA-seq and RT-qPCR data of three genes (*D27*, *CCD8* and *LePS2*). **Suppl. Fig. 4**. PCA of tomato root transcript profiles using RPKM. YP4 and NP4 represent 4 days control P and P starvation treatment in WT, respectively. CCD8.YP4 and CCD8.NP4 represent control P and P starvation treatment in CCD8, respectively. **Suppl. Fig. 5**. Heatmap showing a selection of strongest induced and repressed DEGs in the roots of WT tomato and CCD8 RNAi line under different P starvation treatment times. The RPKM value of the top 10 strongest P starvation induced DEGS (at 2, 3, 4 and 5 days of P starvation) and their repression by P replenishment in WT, and DEGs at 4 days of P starvation in CCD8 RNAi line. **Suppl. Fig. 6**. Secondary metabolism visualization of PS induced DEGs, PS repressed and SL-dependent DEGs with iPath 3.0 [53, 54]. A, secondary metabolite biosynthesis visualization of P starvation repressed DEGs (4 days) in WT. B, secondary metabolite biosynthesis visualization of P starvation repressed and SL-dependent DEGs. **Suppl. Fig. 7**. Expression profiles of steroid and brassinosteroid pathway in the root of tomato under normal P and P starvation. A, schematic representation of steroid biosynthesis and schematic representation of brassinosteroid biosynthesis (from KEGG) [54]. B, hierarchical clustering diagram of brassinosteroid biosynthesis related genes (H1 and H2 represent homolog 1 and 2, respectively). YP4 and NP4 represent tomato WT at 4 days of control P and P starvation, respectively. CCD8.YP4 and CCD8.NP4 represent the CCD8 RNAi line at 4 days of control P and P starvation, respectively. *SMT*, *STEROL 24-C-METHYLTRANSFERASE*; *STE*, *DELTA(7)-STEROL-C5(6)-DESATURASE*; *DWF*, *DELTA (24)-STEROL REDUCTASE*; *SMO*, *C-4Α-STEROL-METHYLOXIDASE2*; *DET*, *STEROID 5-ALPHA-REDUCTASE*. **Suppl. Table 1**. Gene list and go enrichment of 48 DEGs (common P starvation induced DEGs). **Suppl. Table 2**. Summary of Go enrichment of P starvation repressed DEGs. **Suppl. Table 3**. The fold change of 108 DEGs (SL dependent P starvation induced genes) after 4 days P starvation in WT and CCD8. **Suppl. Table 4**. The KO of P starvation induced DEGs. **Suppl. Table 5**. The KO of P starvation repressed DEGs. **Suppl. Table 6**. The KO of 31 DEGs (P starvation repressed and SL dependent DEGs). **Suppl. Table 7**. The KO of 108 DEGs (P starvation induced and SL dependent DEGs). **Suppl. Table 8**. An overview of raw read numbers, and trimming and mapping statistics. **Suppl. Table 9**. Primers used in this study. **Suppl. Data set 1**. The DEGs of 2 days P starvation in wild type. **Suppl. Data set 1. S1**, significant DEGs after 2 days P starvation in wild type. **Suppl. Data set 1. S2**, GO enrichment of 2 days P starvation significantly induced genes in PlantRegMap (*P *value <=0.01). **Suppl. Data set 2**. The DEGs of 3 days P starvation in wild type. **Suppl. Data set 2. S1**, significant DEGs after 3 days P starvation in wild type. **Suppl. Data set 2. S2**, GO enrichment of 3 days P starvation significantly induced genes in PlantRegMap (*P *value <=0.01). **Suppl. Data set 2. S3**, GO enrichment of 3 days P starvation significantly repressed genes in PlantRegMap (*P *value <=0.01). **Suppl. Data set 3**. The DEGs of 4 days P starvation in wild type. **Suppl. Data set 3. S1**, significant DEGs after 4 days P starvation in wild type. **Suppl. Data set 3. S2**, GO enrichment of 4 days P starvation significantly induced genes in PlantRegMap (*P *value <=0.01). **Suppl. Data set 3. S3**, GO enrichment of 4 days P starvation significantly repressed genes in PlantRegMap (*P *value <=0.01). **Suppl. Data set 4**. The DEGs of 5 days P starvation in wild type. **Suppl. Data set 4. S1**, significant DEGs after 5 days P starvation in wild type. **Suppl. Data set 4. S2**, GO enrichment of 5 days P starvation significantly induced genes in PlantRegMap (*P *value <=0.01). **Suppl. Data set 4. S3**, GO enrichment of 5 days P starvation significantly repressed genes in PlantRegMap (*P *value <=0.01). **Suppl. Data set 5**. The DEGs of one day P replenishment in wild type. **Suppl. Data set 5. S1**, significant DEGs of one day P replenishment after 4 days P starvation in wild type. **Suppl. Data set 5. S2**, GO enrichment of one day P replenishment after 4 days P starvation significantly induced genes in PlantRegMap (*P *value <=0.01). **Suppl. Data set 5. S3**, GO enrichment of one day P replenishment after 4 days P starvation significantly repressed genes in PlantRegMap (*P *value <=0.01). **Suppl. Data set 6**. The DEGs of 4 days P starvation in *CCD8* RNAi line. **Suppl. Data set 6. S1**, significant DEGs after 4 days P starvation in *CCD8* RNAi line. **Suppl. Data set 6. S2**, GO enrichment of 4 days P starvation significantly induced genes in PlantRegMap (*P *value <=0.01). **Suppl. Data set 6. S3**, GO enrichment of 4 days P starvation significantly repressed genes in PlantRegMap (*P *value <=0.01). **Suppl. Data set 7**. The DEGs in CCD8 RNAi line compared with wild type under P starvation condition. **Suppl. Data set 7. S1**, significant DEGs in *CCD8* RNAi line compared with wild type after 4 days P starvation. **Suppl. Data set 7. S2**, GO enrichment of significant induced DEGs in *CCD8* RNAi line compared with wild type after 4 days P starvation (*P *value <=0.01). **Suppl. Data set 7. S3**, GO enrichment of significant repressed DEGs in *CCD8* RNAi line compared with wild type after 4 days P starvation (*P *value <=0.01).

## Data Availability

The datasets generated and analyzed during the current study are available in the NCBI Short Read Archive under accession number PRJNA679261 (https://dataview.ncbi.nlm.nih.gov/object/PRJNA679261?reviewer=vs5lk0a94j04c2rgieta1lrlro). The *CCD8* RNAi line 09 tomato seeds were obtained from Plant Physiology of Wageningen University, the Netherland (seed code 121). Experimental research and field studies on the plant material, comply with relevant institutional, national, and international guidelines and legislation.

## Abbreviations

*2-ODD*: *2*-*OXOGLUTARATE-DEPENDENT DIOXYGENASE*
*AAO*: *ARABIDOPSIS ALDEHYDE OXIDASE*
*ACC*: *1-AMINOCYCLOPROPANE-1-CARBOXYLATE*
*AGPL*: *ADP-GLUCOSE PYROPHOSPHORYLASE*
*ALS*: *ABC TRANSPORTER-LIKE*
*AP*: *ACID PHOSPHATASE*
APs: Acid phosphatases
*AtAPs*: *Arabidopsis ACID PHOSPHATASEs*
*BCH*: *BETA-CAROTENE HYDROXYLASE*
*CCD1*-*like*: *CAROTENOID 9*,*10*(*9'*,*10'*)-*CLEAVAGE DIOXYGENASE 1*-*LIKE*
*CCD7*: *CAROTENOID CLEAVAGE DIOXYGENASE 7*
*CCD8*: *CAROTENOID CLEAVAGE DIOXYGENASES 8*
*CCT*: *CTP-PHOSPHOCHOLINE CYTIDYLYLTRANSFERASE*
CDP: Cytidine diphosphate
*CKI*: *CHOLINE KINASE*
*CMK*: *4-DIPHOSPHOCYTIDYL-2-C-METHYL-D-ERYTHRITOL KINASE*
*CPS*: *COPALYL DIPHOSPHATE SYNTHASE*
*D14*: *DWARF 14*
*D27*: *DWARF 27*
DAG: 1:2-Diacylglycerol
DEGs: Differentially expressed genes
*DET*: *STEROID 5*-*ALPHA*-*REDUCTASE*
DGDG: Digalactosyldiacylglycerol
*DGDGS*: *DIGALACTOSYLDIACYLGLYCEROL SYNTHASE*
*DWF*: *DELTA (24)*-*STEROL REDUCTASE*
*DXR*: *1-DEOXY-D-XYLULOSE 5-PHOSPHATE REDUCTOISOMERASE*
*DXS*: *1-DEOXY-D-XYLULOSE-5-PHOSPHATE SYNTHASE*
*ECT*: *CTP-PHOSPHOETHANOLAMINE CYTIDYLYLTRANSFERASE*
*EKI*: *ETHANOLAMINE KINASE*
*GA20OX*: *GIBBERELLIN 20-OXIDASE*
*GA2OX*: *GIBBERELLIN 2-OXIDASE*
*GA3OX*: *GIBBERELLIN 3-OXIDASE*
*GGPPS*: *GERANYLGERANYL PYROPHOSPHATE SYNTHETASE*
Glc1P: Glucose 1-phosphate
GO: Gene ontology
*GPAT*: *GLYCEROL-PHOSPHATE ACYLTRANSFERASE*
*GPDE*: *GLYCEROPHOSPHODIESTERASE*
*GST*: *GLUTATHIONE-S-TRANSFERASE*
*GT8*: *GLYCOSYLTRANSFERASE FAMILY 8*
HCA: Hierarchical clustering analysis
*HDR*: *4-HYDROXY-3-METHYLBUT-2-ENYL DIPHOSPHATE REDUCTASE*
*IPS1*: *INDUCED BY PHOSPHATE STARVATION1*
*KAO*: *ENT-KAURENOIC ACID OXIDASE*
*KO*: *ENT-KAURENE OXIDASE*
*KS*: *ENT-KAURENE SYNTHASE*
*LaSAP2*: White lupin (*Lupinus albus*) *SECRETORY ACID PHOSPHATASE 2*
*LCY*: *LYCOPENE BETA CYCLASE*
*LePS2*: (*Lycopersicon esculentum*) *phosphate starvation*-*induced gene*
*MAX1*: *MORE AXILLARY GROWTH 1*
*MAX2*: *MORE AXILLARY GROWTH 2*
*MCT*: *2-C-METHYL-D-ERYTHRITOL 4-PHOSPHATE CYTIDYLYLTRANSFERASE*
*MDS*: *2-C-METHYL-D-ERYTHRITOL 2:4-CYCLODIPHOSPHATE SYNTHASE*
*MDS*: *4-HYDROXY-3-METHYLBUT-2-EN-1-YL DIPHOSPHATE SYNTHASE*
MGDG: Monogalactosyldiacylglycerol
*MGDGS*: *MONOGALACTOSYLDIACYLGLYCEROL SYNTHASE*
*NCED*: *EPOXYCAROTENOID DIOXYGENASE*
NP: Half-strength Hoagland solution without P
*NSY*: *NEOXANTHIN SYNTHASE*
P: Phosphorus
PAP: *PURPLE ACID PHOSPHATASE*
PC: Phosphatidylcholine
PCA: Principal component analysis
*PDS*: *PHYTOENE DESATURASE*
PE: Phosphatidylethanolamine
*PEAMT*: *PHOSPHOETHANOLAMINE N-METHYLTRANSFERASE*
*PEAMT*: *PHOSPHORYLETHANOLAMINE N-METHYLTRANSFERASE*
*PFK*: *PHOSPHOFRUCTOKINASE*
*PGM*: *PHOSPHOGLYCERATE MUTASE*
*PHO2*: *PHOSPHATE2*
*PHT1*: *PHOSPHATE TRANSPORTER 1*
*PLD*: *PHOSPHOLIPID DEGRADATION*
*PLMT*: *PHOSPHOLIPID N-METHYLTRANSFERASE*
PPi: Pyrophosphate
*PSY*: *PHYTOENE SYNTHASE*
RP: P replenishment after P deficiency
RPKM: Reads per kilobase of transcript per million mapped reads
*SAUR*: *AUXIN RESPONSIVE*
SLs: Strigolactones
*SMO*: *C*-*4Α*-*STEROL*-*METHYLOXIDASE2*
*SMT*: *STEROL 24*-*C*-*METHYLTRANSFERASE*
*SMXL 8*: *SUPPRESSOR OF MAX2 1-LIKE 8*
*SMXL6*: *SUPPRESSOR OF MAX2 1-LIKE 6*
*SPS*: *SUCROSE PHOSPHATE SYNTHESIS*
SQ: Sulfoquinovose
SQDG: Sulfoquinovosyldiacylglycerol
*SQDGS*: *SULFOQUINOVOSYLDIACYLGLYCEROL SYNTHASE*
*SSL*: *STRICTOSIDINE SYNTHASE*-*LIKE*
*STE*: *DELTA(7)*-*STEROL*-*C5(6)*-*DESATURASE*
*UGT*: *UDP*-*GLUCOSYLTRANSFERASE*
UTP: Uridine-5′-triphosphate
YP: Half-strength Hoagland solution with P
*ZDS*: *ZETA-CAROTENE DESATURASE*
*ZEP*: *ZEAXANTHIN EPOXIDASE*
*2-ODD*: *2*-*OXOGLUTARATE-DEPENDENT DIOXYGENASE*
*AAO*: *ARABIDOPSIS ALDEHYDE OXIDASE*
*ACC*: *1-AMINOCYCLOPROPANE-1-CARBOXYLATE*
*AGPL*: *ADP-GLUCOSE PYROPHOSPHORYLASE*
*ALS*: *ABC TRANSPORTER-LIKE*
*AP*: *ACID PHOSPHATASE*
APs: Acid phosphatases
*AtAPs*: *Arabidopsis ACID PHOSPHATASEs*
*BCH*: *BETA-CAROTENE HYDROXYLASE*
*CCD1*-*like*: *CAROTENOID 9*,*10*(*9'*,*10'*)-*CLEAVAGE DIOXYGENASE 1*-*LIKE*
*CCD7*: *CAROTENOID CLEAVAGE DIOXYGENASE 7*
*CCD8*: *CAROTENOID CLEAVAGE DIOXYGENASES 8*
*CCT*: *CTP-PHOSPHOCHOLINE CYTIDYLYLTRANSFERASE*
CDP: Cytidine diphosphate
*CKI*: *CHOLINE KINASE*
*CMK*: *4-DIPHOSPHOCYTIDYL-2-C-METHYL-D-ERYTHRITOL KINASE*
*CPS*: *COPALYL DIPHOSPHATE SYNTHASE*
*D14*: *DWARF 14*
*D27*: *DWARF 27*
DAG: 1:2-Diacylglycerol
DEGs: Differentially expressed genes
*DET*: *STEROID 5*-*ALPHA*-*REDUCTASE*
DGDG: Digalactosyldiacylglycerol
*DGDGS*: *DIGALACTOSYLDIACYLGLYCEROL SYNTHASE*
*DWF*: *DELTA (24)*-*STEROL REDUCTASE*
*DXR*: *1-DEOXY-D-XYLULOSE 5-PHOSPHATE REDUCTOISOMERASE*
*DXS*: *1-DEOXY-D-XYLULOSE-5-PHOSPHATE SYNTHASE*
*ECT*: *CTP-PHOSPHOETHANOLAMINE CYTIDYLYLTRANSFERASE*
*EKI*: *ETHANOLAMINE KINASE*
*GA20OX*: *GIBBERELLIN 20-OXIDASE*
*GA2OX*: *GIBBERELLIN 2-OXIDASE*
*GA3OX*: *GIBBERELLIN 3-OXIDASE*
*GGPPS*: *GERANYLGERANYL PYROPHOSPHATE SYNTHETASE*
Glc1P: Glucose 1-phosphate
GO: Gene ontology
*GPAT*: *GLYCEROL-PHOSPHATE ACYLTRANSFERASE*
*GPDE*: *GLYCEROPHOSPHODIESTERASE*
*GST*: *GLUTATHIONE-S-TRANSFERASE*
*GT8*: *GLYCOSYLTRANSFERASE FAMILY 8*
HCA: Hierarchical clustering analysis
*HDR*: *4-HYDROXY-3-METHYLBUT-2-ENYL DIPHOSPHATE REDUCTASE*
*IPS1*: *INDUCED BY PHOSPHATE STARVATION1*
*KAO*: *ENT-KAURENOIC ACID OXIDASE*
*KO*: *ENT-KAURENE OXIDASE*
*KS*: *ENT-KAURENE SYNTHASE*
*LaSAP2*: White lupin (*Lupinus albus*) *SECRETORY ACID PHOSPHATASE 2*
*LCY*: *LYCOPENE BETA CYCLASE*
*LePS2*: (*Lycopersicon esculentum*) *phosphate starvation*-*induced gene*
*MAX1*: *MORE AXILLARY GROWTH 1*
*MAX2*: *MORE AXILLARY GROWTH 2*
*MCT*: *2-C-METHYL-D-ERYTHRITOL 4-PHOSPHATE CYTIDYLYLTRANSFERASE*
*MDS*: *2-C-METHYL-D-ERYTHRITOL 2:4-CYCLODIPHOSPHATE SYNTHASE*
*MDS*: *4-HYDROXY-3-METHYLBUT-2-EN-1-YL DIPHOSPHATE SYNTHASE*
MGDG: Monogalactosyldiacylglycerol
*MGDGS*: *MONOGALACTOSYLDIACYLGLYCEROL SYNTHASE*
*NCED*: *EPOXYCAROTENOID DIOXYGENASE*
NP: Half-strength Hoagland solution without P
*NSY*: *NEOXANTHIN SYNTHASE*
P: Phosphorus
PAP: *PURPLE ACID PHOSPHATASE*
PC: Phosphatidylcholine
PCA: Principal component analysis
*PDS*: *PHYTOENE DESATURASE*
PE: Phosphatidylethanolamine
*PEAMT*: *PHOSPHOETHANOLAMINE N-METHYLTRANSFERASE*
*PEAMT*: *PHOSPHORYLETHANOLAMINE N-METHYLTRANSFERASE*
*PFK*: *PHOSPHOFRUCTOKINASE*
*PGM*: *PHOSPHOGLYCERATE MUTASE*
*PHO2*: *PHOSPHATE2*
*PHT1*: *PHOSPHATE TRANSPORTER 1*
*PLD*: *PHOSPHOLIPID DEGRADATION*
*PLMT*: *PHOSPHOLIPID N-METHYLTRANSFERASE*
PPi: Pyrophosphate
*PSY*: *PHYTOENE SYNTHASE*
RP: P replenishment after P deficiency
RPKM: Reads per kilobase of transcript per million mapped reads
*SAUR*: *AUXIN RESPONSIVE*
SLs: Strigolactones
*SMO*: *C*-*4Α*-*STEROL*-*METHYLOXIDASE2*
*SMT*: *STEROL 24*-*C*-*METHYLTRANSFERASE*
*SMXL 8*: *SUPPRESSOR OF MAX2 1-LIKE 8*
*SMXL6*: *SUPPRESSOR OF MAX2 1-LIKE 6*
*SPS*: *SUCROSE PHOSPHATE SYNTHESIS*
SQ: Sulfoquinovose
SQDG: Sulfoquinovosyldiacylglycerol
*SQDGS*: *SULFOQUINOVOSYLDIACYLGLYCEROL SYNTHASE*
*SSL*: *STRICTOSIDINE SYNTHASE*-*LIKE*
*STE*: *DELTA(7)*-*STEROL*-*C5(6)*-*DESATURASE*
*UGT*: *UDP*-*GLUCOSYLTRANSFERASE*
UTP: Uridine-5′-triphosphate
YP: Half-strength Hoagland solution with P
*ZDS*: *ZETA-CAROTENE DESATURASE*
*ZEP*: *ZEAXANTHIN EPOXIDASE*
