# Supplementary material for: The role of strigolactones in P deficiency induced transcriptional changes in tomato roots
Source: BMC Plant Biol. 2021 Jul 23;21:349. doi: 10.1186/s12870-021-03124-0 (PMC8299696; doi:10.1186/s12870-021-03124-0)
Supplement: Supplementary file 1 — Additional file 1:Suppl. Fig. 1. Experimental design of the P starvation RNAseq experiment. Suppl. Fig. 2. KEGG pathway enrichment for induced (A) and repressed (B) DEGs in tomato roots upon P starvation and P replenishment. Suppl. Fig. 3. Heatmap showing fold change of SL related genes in RNAseq dataset and RT-qPCR validation of SL biosynthetic and P starvation marker genes. A, heatmap showing fold change of genes involved in the SL biosynthetic and signaling pathway under P starvation for different time periods and P replenishment in WT tomato and CCD8 RNAi line. A fold change in bold indicates significance (P<0.05). B-D, relative expression of D27 (B), CCD8 (C) and LePS2 (D) upon P starvation (and P replenishment) (n = 3). The gene expression level in 2-day control wild‐type plants (YP2) was set to 1. Error bars represent standard error of the mean. **, 0.01>P; *, 0.01 < P < 0.05; NG, not significant. E, comparison of RT-qPCR and RNA-seq data. A Pearson correlation coefficient of 0.9761 (P < 0.01) is observed between the RNA-seq and RT-qPCR data of three genes (D27, CCD8 and LePS2). Suppl. Fig. 4. PCA of tomato root transcript profiles using RPKM. YP4 and NP4 represent 4 days control P and P starvation treatment in WT, respectively. CCD8.YP4 and CCD8.NP4 represent control P and P starvation treatment in CCD8, respectively. Suppl. Fig. 5. Heatmap showing a selection of strongest induced and repressed DEGs in the roots of WT tomato and CCD8 RNAi line under different P starvation treatment times. The RPKM value of the top 10 strongest P starvation induced DEGS (at 2, 3, 4 and 5 days of P starvation) and their repression by P replenishment in WT, and DEGs at 4 days of P starvation in CCD8 RNAi line. Suppl. Fig. 6. Secondary metabolism visualization of PS induced DEGs, PS repressed and SL-dependent DEGs with iPath 3.0 [53, 54]. A, secondary metabolite biosynthesis visualization of P starvation repressed DEGs (4 days) in WT. B, secondary metabolite biosynthesis visuali [file 12870_2021_3124_MOESM1_ESM.zip › Supplementary Information_2nd revised version.docx]

**The role of strigolactones in P deficiency induced transcriptional changes in tomato roots**

Yanting Wang^1^, Hernando G. Suárez Duran^2^, Jan C. van Haarst^3^, Elio G. W. M. Schijlen^3^, Carolien Ruyter-Spira^4^, Marnix H. Medema^2^, Lemeng Dong^1^, Harro J. Bouwmeester^1*^

^1^Plant Hormone Biology group, Swammerdam Institute for Life Sciences, University of Amsterdam, Amsterdam, The Netherlands

^2^Bioinformatics Group, Wageningen University and Research, Wageningen, The Netherlands

^3^Business Unit Bioscience, Plant Research International, Wageningen, The Netherlands

^4^Laboratory of Plant Physiology, Wageningen University and Research, Wageningen, The Netherlands

* Correspondence: [H.J.Bouwmeester@uva.nl](mailto:H.J.Bouwmeester@uva.nl)

**Supplementary Information**

Suppl. Fig. 1-7

**
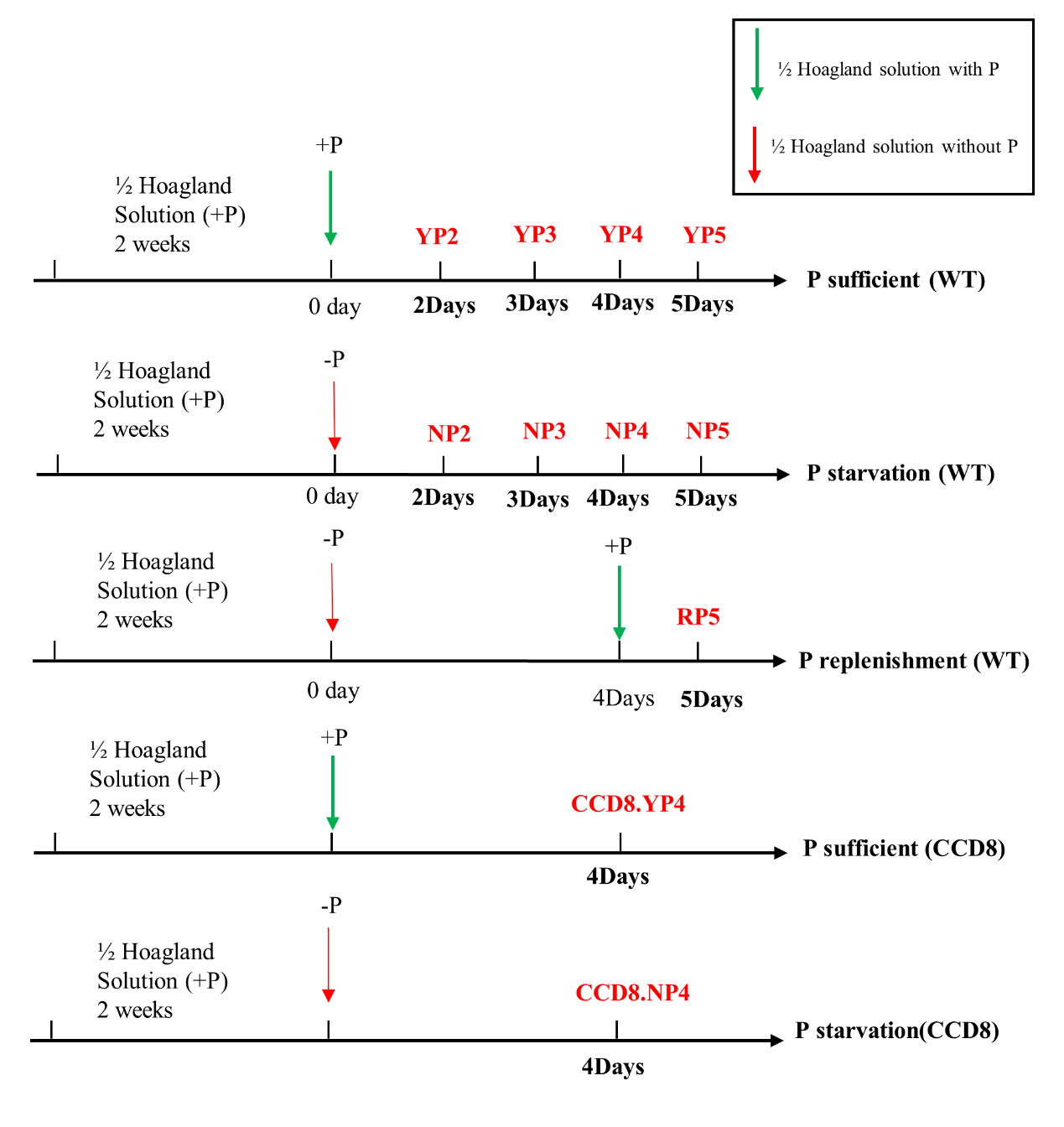
Suppl. Fig. 1. Experimental design of the P starvation RNAseq experiment.**

**
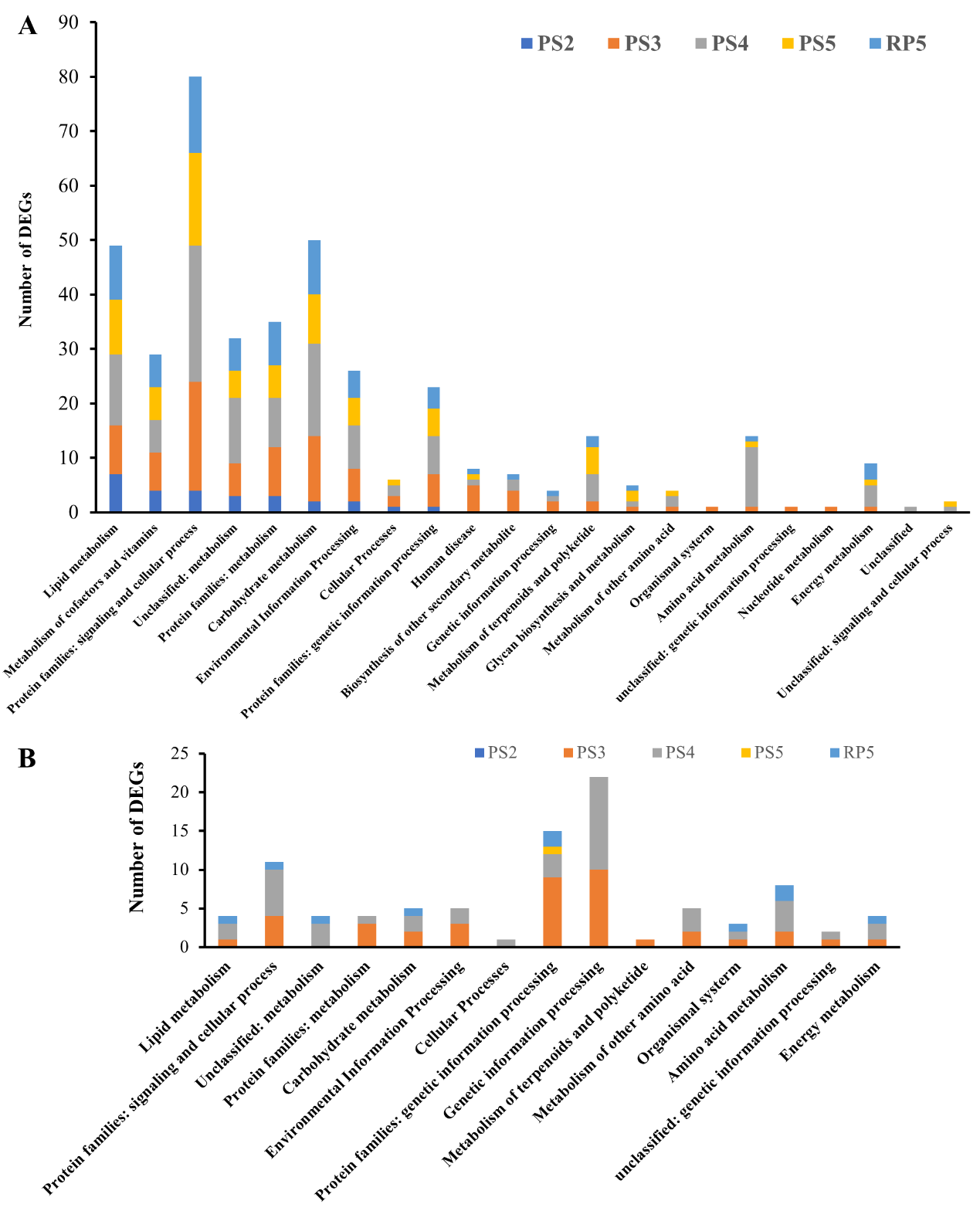
**

**Suppl. Fig. 2. KEGG pathway enrichment for induced (A) and repressed (B) DEGs in tomato roots upon P starvation and P replenishment.**

**
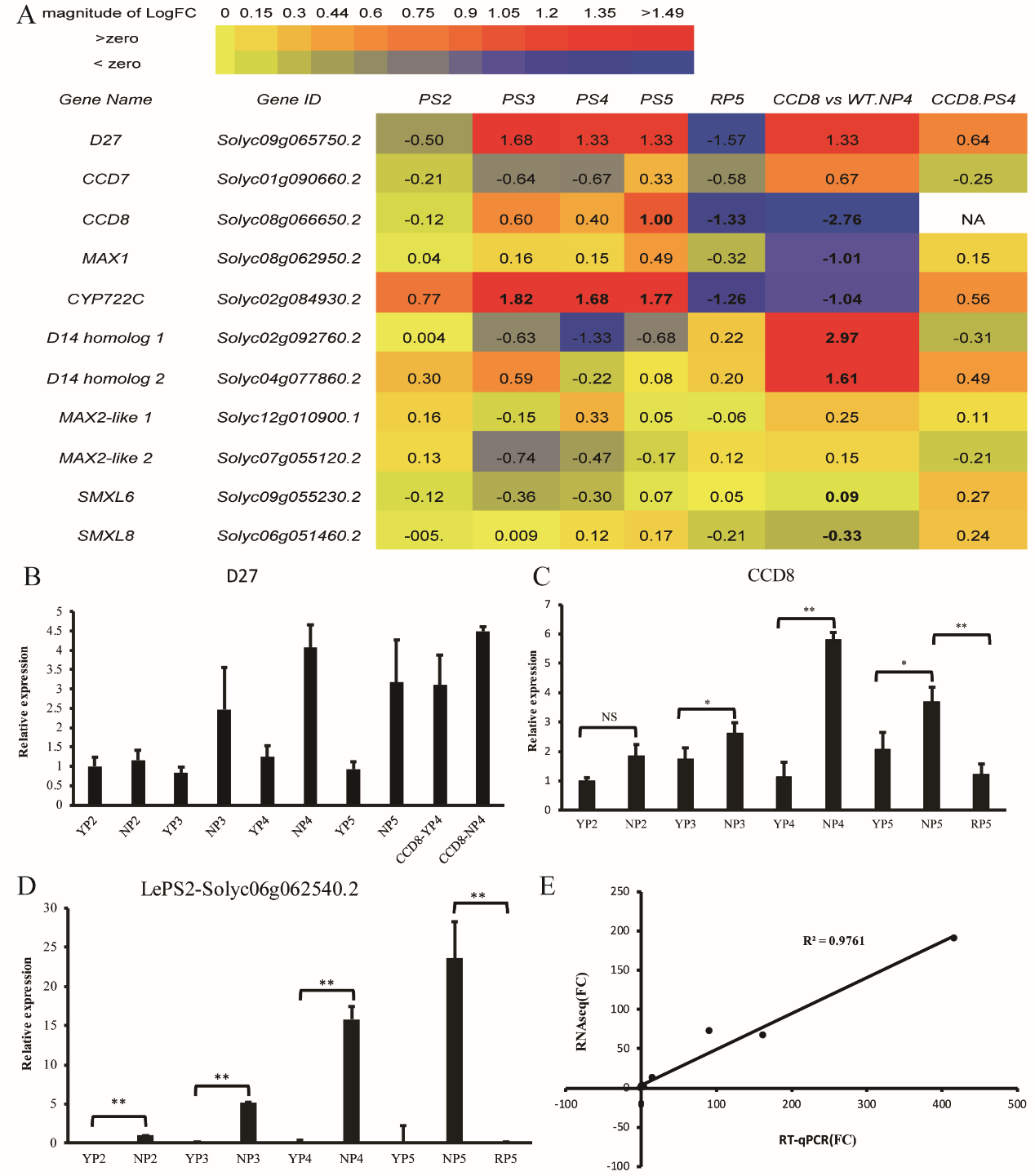
**

**Suppl. Fig. 3.** **Heatmap showing fold change of SL related genes in RNAseq dataset and RT-qPCR validation of SL biosynthetic and P starvation marker genes. A, heatmap showing fold change of genes involved in the SL biosynthetic and signaling pathway under P starvation for different time periods and P replenishment in WT tomato and CCD8 RNAi line. A fold change in bold indicates significance (P<0.05). B-D, relative expression of *D27* (B), *CCD8* (C) and *LePS2* (D) upon P starvation (and P replenishment) (n = 3). The gene expression level in 2-day control wild‐type plants (YP2) was set to 1. Error bars represent standard error of the mean. **, 0.01>P; *, 0.01 < P < 0.05; NG, not significant. E, comparison of RT-qPCR and RNA-seq data. A Pearson correlation coefficient of 0.9761 (P < 0.01) is observed between the RNA-seq and RT-qPCR data of three genes (*D27*, *CCD8* and *LePS2*).**

**
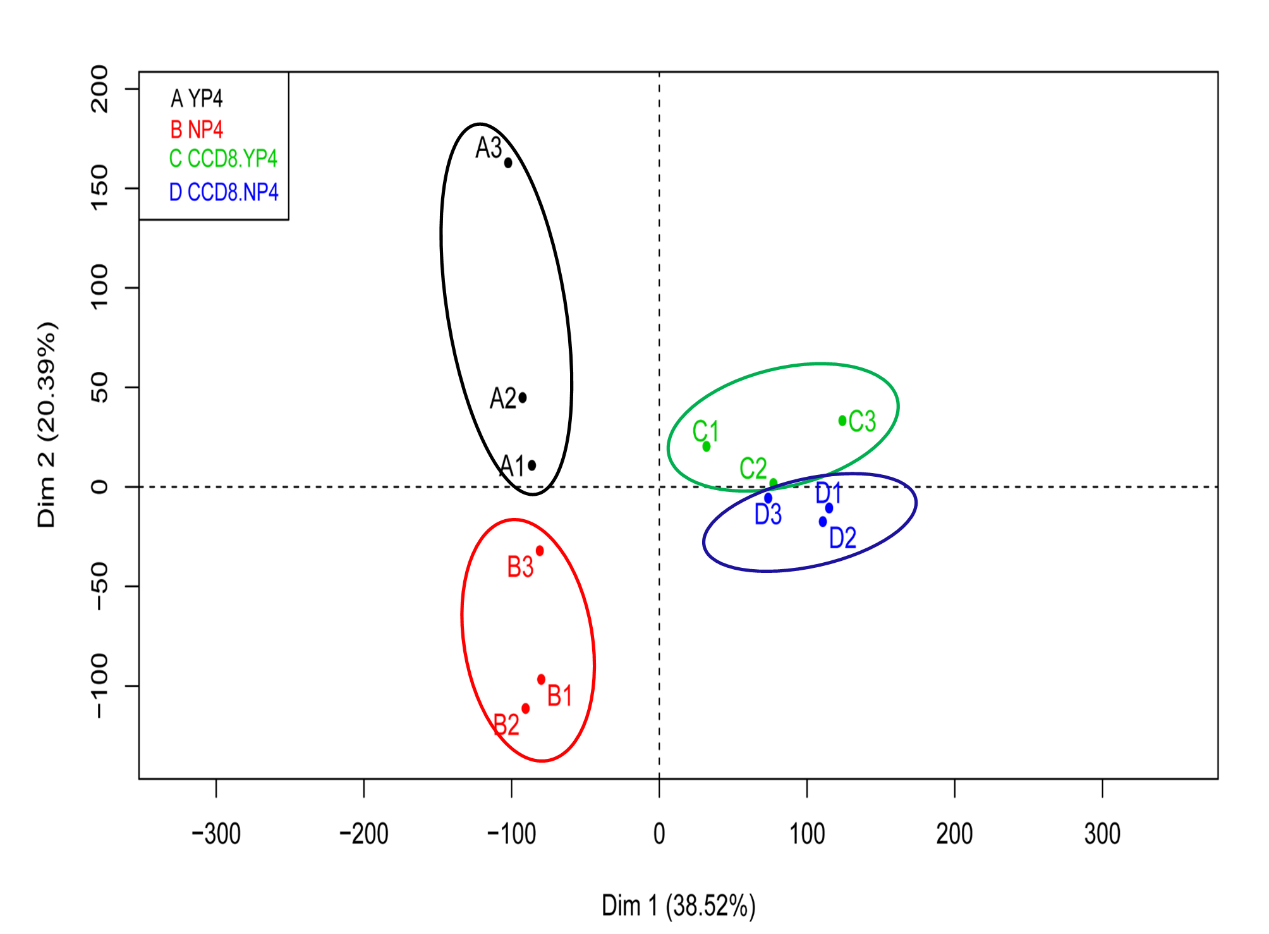
**

**Suppl. Fig. 4.** **PCA of tomato root transcript profiles using RPKM.** YP4 and NP4 represent 4 days control P and P starvation treatment in WT, respectively. CCD8.YP4 and CCD8.NP4 represent control P and P starvation treatment in CCD8, respectively.

**
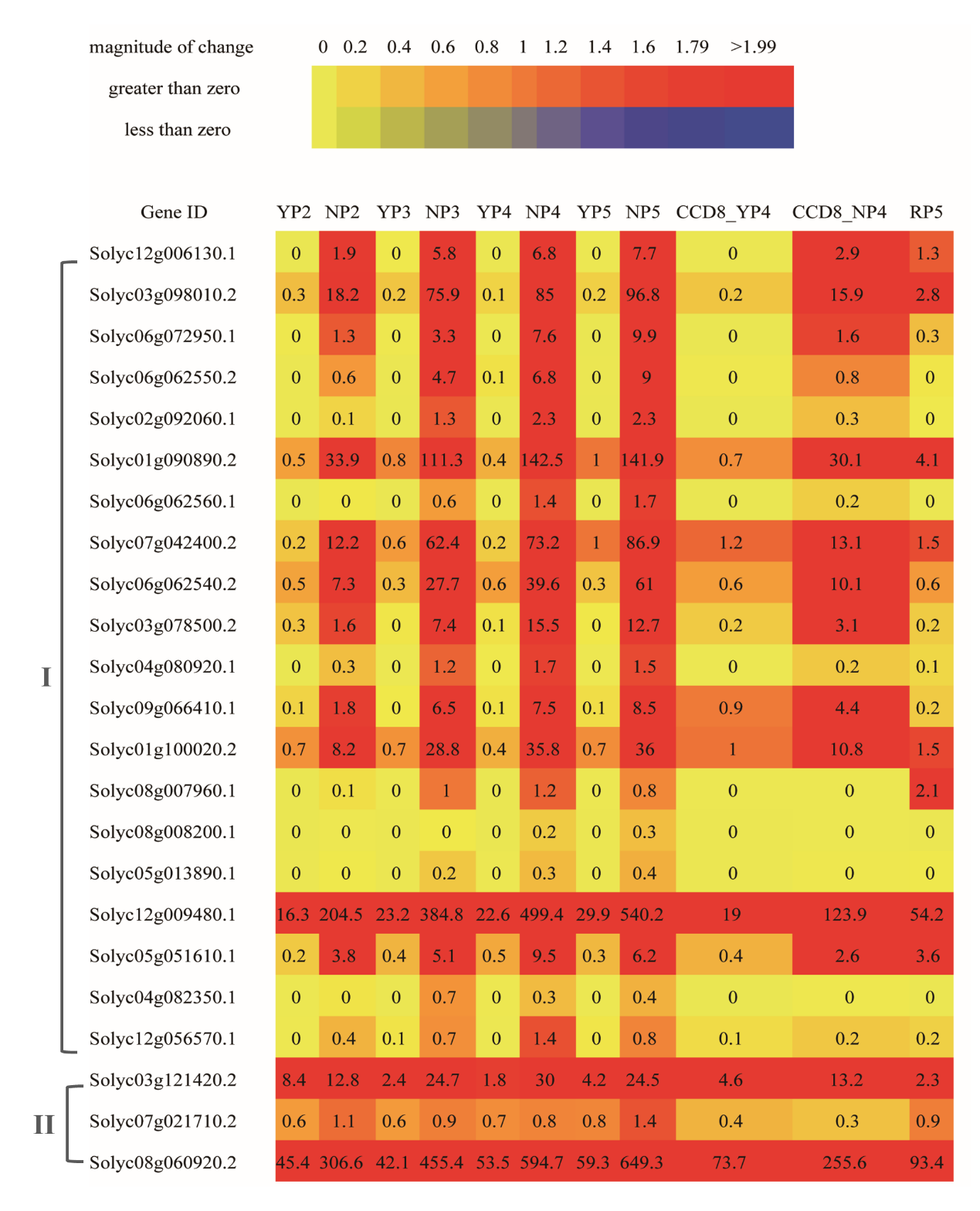
Suppl. Fig. 5. Heatmap showing a selection of strongest induced and repressed DEGs in the roots of WT tomato and CCD8 RNAi line under different P starvation treatment times.** The RPKM value of the top 10 strongest P starvation induced DEGS (at 2, 3, 4 and 5 days of P starvation) and their repression by P replenishment in WT, and DEGs at 4 days of P starvation in CCD8 RNAi line.

**
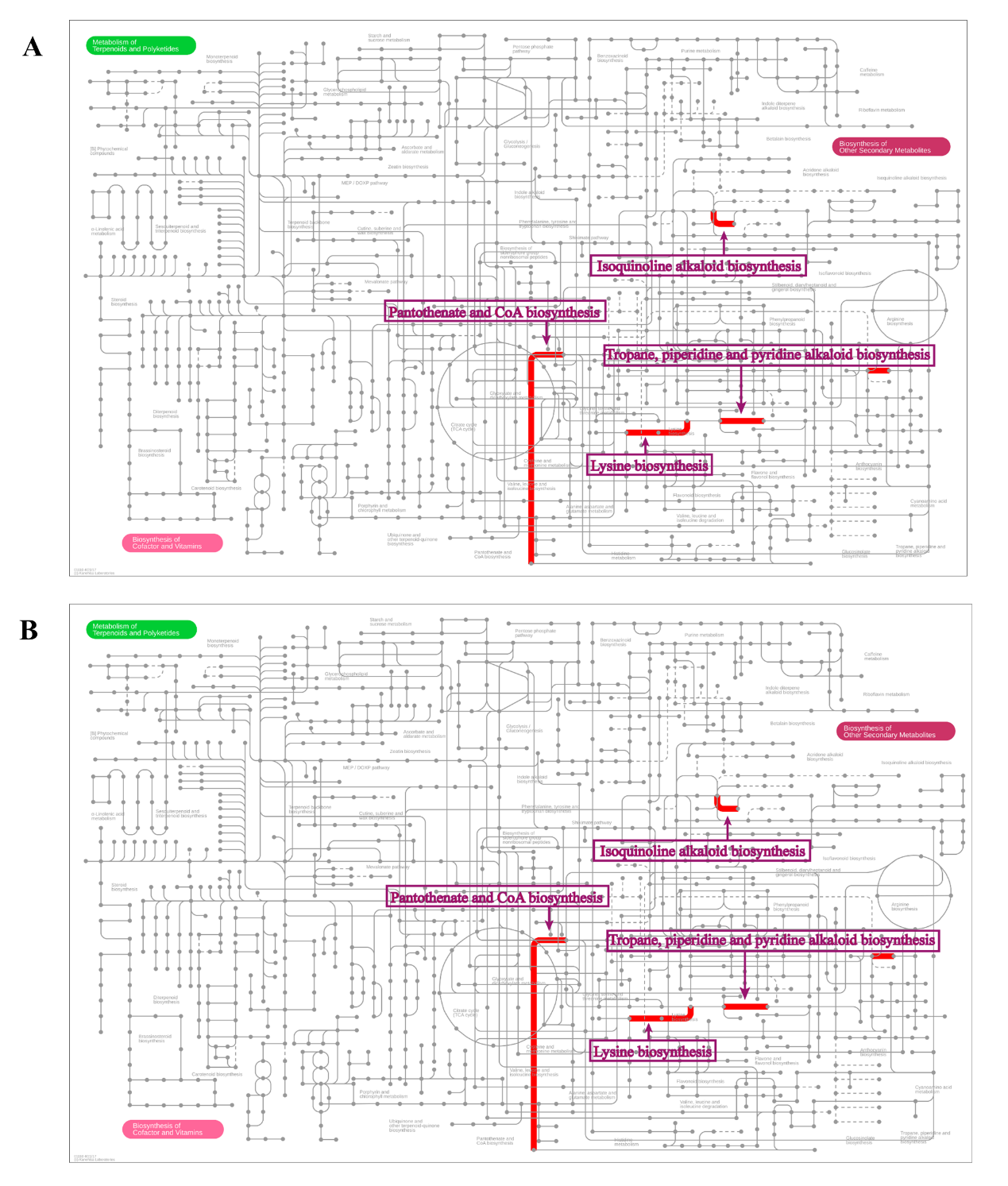
Suppl. Fig. 6. Secondary metabolism visualization of PS induced DEGs, PS repressed and SL-dependent DEGs with iPath 3.0 [**[**1**](#_ENREF_1)**,** [**2**](#_ENREF_2)**].** A, secondary metabolite biosynthesis visualization of P starvation repressed DEGs (4 days) in WT. B, secondary metabolite biosynthesis visualization of P starvation repressed and SL-dependent DEGs.

**
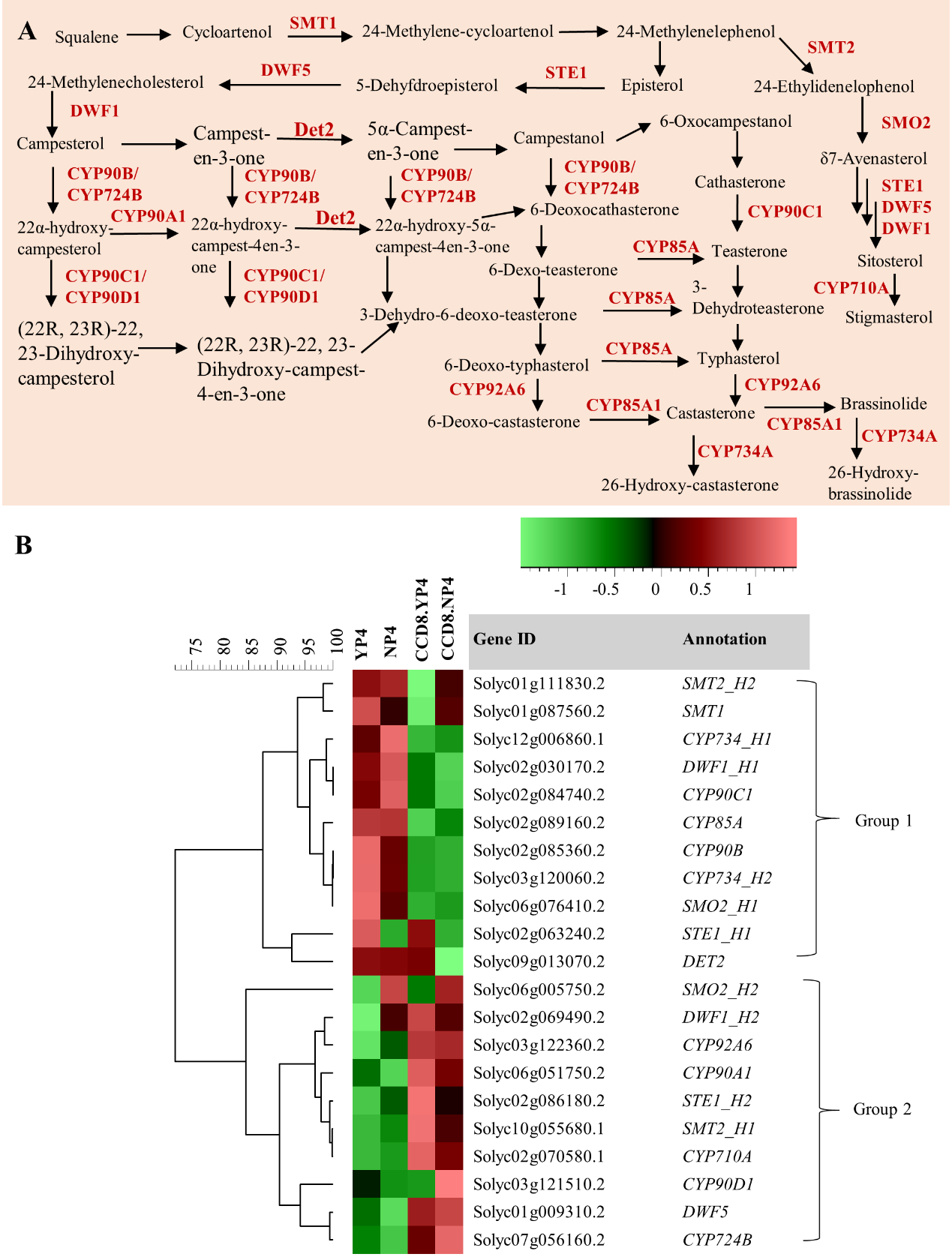
Suppl. Fig. 7. Expression profiles of steroid and brassinosteroid pathway in the root of tomato under normal P and P starvation.** A, schematic representation of steroid biosynthesis and schematic representation of brassinosteroid biosynthesis (from KEGG) [[1](#_ENREF_1)]. B, hierarchical clustering diagram of brassinosteroid biosynthesis related genes (H1 and H2 represent homolog 1 and 2, respectively). YP4 and NP4 represent tomato WT at 4 days of control P and P starvation, respectively. CCD8.YP4 and CCD8.NP4 represent the CCD8 RNAi line at 4 days of control P and P starvation, respectively. *SMT*, *STEROL 24-C-METHYLTRANSFERASE*; *STE*, *DELTA(7)-STEROL-C5(6)-DESATURASE*; *DWF*, *DELTA (24)-STEROL REDUCTASE*; *SMO*, *C-4Α-STEROL-METHYLOXIDASE2*; *DET*, *STEROID 5-ALPHA-REDUCTASE*.

**Suppl. Table 1- Suppl. Table 9**

**Suppl. Table 1.** Gene list and go enrichment of 48 DEGs (common P starvation induced DEGs).

**Suppl. Table 2.** Summary of Go enrichment of P starvation repressed DEGs.

**Suppl. Table 3.** The fold change of 108 DEGs (SL dependent P starvation induced genes) after 4 days P starvation in WT and CCD8.

**Suppl. Table 4.** The KO of P starvation induced DEGs.

**Suppl. Table 5.** The KO of P starvation repressed DEGs.

**Suppl. Table 6.** The KO of 31 DEGs (P starvation repressed and SL dependent DEGs).

**Suppl. Table 7.** The KO of 108 DEGs (P starvation induced and SL dependent DEGs).

**Suppl. Table 8.** An overview of raw read numbers, and trimming and mapping statistics.

**Suppl. Table 9.** Primers used in this study.

**Suppl. Data set 1. The DEGs of 2 days P starvation in wild type.**

Suppl. Data set 1. S1, significant DEGs after 2 days P starvation in wild type.

Suppl. Data set 1. S2, GO enrichment of 2 days P starvation significantly induced genes in PlantRegMap (P value <=0.01).

**Suppl. Data set 2. The DEGs of 3 days P starvation in wild type.**

Suppl. Data set 2. S1, significant DEGs after 3 days P starvation in wild type.

Suppl. Data set 2. S2, GO enrichment of 3 days P starvation significantly induced genes in PlantRegMap (P value <=0.01).

Suppl. Data set 2. S3, GO enrichment of 3 days P starvation significantly repressed genes in PlantRegMap (P value <=0.01).

**Suppl. Data set 3. The DEGs of 4 days P starvation in wild type.**

Suppl. Data set 3. S1, significant DEGs after 4 days P starvation in wild type.

Suppl. Data set 3. S2, GO enrichment of 4 days P starvation significantly induced genes in PlantRegMap (P value <=0.01).

Suppl. Data set 3. S3, GO enrichment of 4 days P starvation significantly repressed genes in PlantRegMap (P value <=0.01).

**Suppl. Data set 4. The DEGs of 5 days P starvation in wild type.**

Suppl. Data set 4. S1, significant DEGs after 5 days P starvation in wild type.

Suppl. Data set 4. S2, GO enrichment of 5 days P starvation significantly induced genes in PlantRegMap (P value <=0.01).

Suppl. Data set 4. S3, GO enrichment of 5 days P starvation significantly repressed genes in PlantRegMap (P value <=0.01).

**Suppl. Data set 5. The DEGs of one day P replenishment in wild type.**

Suppl. Data set 5. S1, significant DEGs of one day P replenishment after 4 days P starvation in wild type.

Suppl. Data set 5. S2, GO enrichment of one day P replenishment after 4 days P starvation significantly induced genes in PlantRegMap (P value <=0.01).

Suppl. Data set 5. S3, GO enrichment of one day P replenishment after 4 days P starvation significantly repressed genes in PlantRegMap (P value <=0.01).

**Suppl. Data set 6. The DEGs of 4 days P starvation in *CCD8* RNAi line.**

Suppl. Data set 6. S1, significant DEGs after 4 days P starvation in *CCD8* RNAi line.

Suppl. Data set 6. S2, GO enrichment of 4 days P starvation significantly induced genes in PlantRegMap (P value <=0.01).

Suppl. Data set 6. S3, GO enrichment of 4 days P starvation significantly repressed genes in PlantRegMap (P value <=0.01).

**Suppl. Data set 7. The DEGs in CCD8 RNAi line compared with wild type under P starvation condition.**

Suppl. Data set 7. S1, significant DEGs in *CCD8* RNAi line compared with wild type after 4 days P starvation.

Suppl. Data set 7. S2, GO enrichment of significant induced DEGs in *CCD8* RNAi line compared with wild type after 4 days P starvation (P value <=0.01).

Suppl. Data set 7. S3, GO enrichment of significant repressed DEGs in *CCD8* RNAi line compared with wild type after 4 days P starvation (P value <=0.01).

1. Kanehisa M: Toward understanding the origin and evolution of cellular organisms. *Protein science : a publication of the Protein Society* 2019, 28(11):1947-1951.

2. Darzi Y, Letunic I, Bork P, Yamada T: iPath3.0: interactive pathways explorer v3. *Nucleic Acids Res* 2018, 46(W1):W510-w513.
